# Deep-coverage whole genome sequences and blood lipids among 16,324 individuals

**DOI:** 10.1038/s41467-018-05747-8

**Published:** 2018-08-23

**Authors:** Pradeep Natarajan, Gina M. Peloso, Seyedeh Maryam Zekavat, May Montasser, Andrea Ganna, Mark Chaffin, Amit V. Khera, Wei Zhou, Jonathan M. Bloom, Jesse M. Engreitz, Jason Ernst, Jeffrey R. O’Connell, Sanni E. Ruotsalainen, Maris Alver, Ani Manichaikul, W. Craig Johnson, James A. Perry, Timothy Poterba, Cotton Seed, Ida L. Surakka, Tonu Esko, Samuli Ripatti, Veikko Salomaa, Adolfo Correa, Ramachandran S. Vasan, Manolis Kellis, Benjamin M. Neale, Eric S. Lander, Goncalo Abecasis, Braxton Mitchell, Stephen S. Rich, James G. Wilson, L. Adrienne Cupples, Jerome I. Rotter, Cristen J. Willer, Sekar Kathiresan, NHLBI TOPMed Lipids Working Group, Namiko Abe, Christine Albert, Nicholette (Nichole) Palmer Allred, Laura Almasy, Alvaro Alonso, Seth Ament, Peter Anderson, Pramod Anugu, Deborah Applebaum-Bowden, Dan Arking, Donna K Arnett, Allison Ashley-Koch, Stella Aslibekyan, Tim Assimes, Paul Auer, Dimitrios Avramopoulos, John Barnard, Kathleen Barnes, R. Graham Barr, Emily Barron-Casella, Terri Beaty, Diane Becker, Lewis Becker, Rebecca Beer, Ferdouse Begum, Amber Beitelshees, Emelia Benjamin, Marcos Bezerra, Larry Bielak, Joshua Bis, Thomas Blackwell, John Blangero, Eric Boerwinkle, Ingrid Borecki, Russell Bowler, Jennifer Brody, Ulrich Broeckel, Jai Broome, Karen Bunting, Esteban Burchard, Jonathan Cardwell, Cara Carty, Richard Casaburi, James Casella, Christy Chang, Daniel Chasman, Sameer Chavan, Bo-Juen Chen, Wei-Min Chen, Yii-Der Ida Chen, Michael Cho, Seung Hoan Choi, Lee-Ming Chuang, Mina Chung, Elaine Cornell, Carolyn Crandall, James Crapo, Joanne Curran, Jeffrey Curtis, Brian Custer, Coleen Damcott, Dawood Darbar, Sayantan Das, Sean David, Colleen Davis, Michelle Daya, Mariza de Andrade, Michael DeBaun, Ranjan Deka, Dawn DeMeo, Scott Devine, Ron Do, Qing Duan, Ravi Duggirala, Peter Durda, Susan Dutcher, Charles Eaton, Lynette Ekunwe, Patrick Ellinor, Leslie Emery, Charles Farber, Leanna Farnam, Tasha Fingerlin, Matthew Flickinger, Myriam Fornage, Nora Franceschini, Mao Fu, Malia Fullerton, Lucinda Fulton, Stacey Gabriel, Weiniu Gan, Yan Gao, Margery Gass, Bruce Gelb, Xiaoqi (Priscilla) Geng, Soren Germer, Chris Gignoux, Mark Gladwin, David Glahn, Stephanie Gogarten, Da-Wei Gong, Harald Goring, C. Charles Gu, Yue Guan, Xiuqing Guo, Jeff Haessler, Michael Hall, Daniel Harris, Nicola Hawley, Jiang He, Ben Heavner, Susan Heckbert, Ryan Hernandez, David Herrington, Craig Hersh, Bertha Hidalgo, James Hixson, John Hokanson, Elliott Hong, Karin Hoth, Chao (Agnes) Hsiung, Haley Huston, Chii Min Hwu, Marguerite Ryan Irvin, Rebecca Jackson, Deepti Jain, Cashell Jaquish, Min A Jhun, Jill Johnsen, Andrew Johnson, Rich Johnston, Kimberly Jones, Hyun Min Kang, Robert Kaplan, Sharon Kardia, Laura Kaufman, Shannon Kelly, Eimear Kenny, Michael Kessler, Alyna Khan, Greg Kinney, Barbara Konkle, Charles Kooperberg, Holly Kramer, Stephanie Krauter, Christoph Lange, Ethan Lange, Leslie Lange, Cathy Laurie, Cecelia Laurie, Meryl LeBoff, Seunggeun Shawn Lee, Wen-Jane Lee, Jonathon LeFaive, David Levine, Dan Levy, Joshua Lewis, Yun Li, Honghuang Lin, Keng Han Lin, Simin Liu, Yongmei Liu, Ruth Loos, Steven Lubitz, Kathryn Lunetta, James Luo, Michael Mahaney, Barry Make, JoAnn Manson, Lauren Margolin, Lisa Martin, Susan Mathai, Rasika Mathias, Patrick McArdle, Merry-Lynn McDonald, Sean McFarland, Stephen McGarvey, Hao Mei, Deborah A Meyers, Julie Mikulla, Nancy Min, Mollie Minear, Ryan L Minster, Solomon Musani, Stanford Mwasongwe, Josyf C Mychaleckyj, Girish Nadkarni, Rakhi Naik, Sergei Nekhai, Deborah Nickerson, Kari North, Tim O’Connor, Heather Ochs-Balcom, James Pankow, George Papanicolaou, Margaret Parker, Afshin Parsa, Sara Penchev, Juan Manuel Peralta, Marco Perez, Ulrike Peters, Patricia Peyser, Larry Phillips, Sam Phillips, Toni Pollin, Wendy Post, Julia Powers Becker, Meher Preethi Boorgula, Michael Preuss, Dmitry Prokopenko, Bruce Psaty, Pankaj Qasba, Dandi Qiao, Zhaohui Qin, Nicholas Rafaels, Laura Raffield, D. C. Rao, Laura Rasmussen-Torvik, Aakrosh Ratan, Susan Redline, Robert Reed, Elizabeth Regan, Alex Reiner, Ken Rice, Dan Roden, Carolina Roselli, Ingo Ruczinski, Pamela Russell, Sarah Ruuska, Kathleen Ryan, Phuwanat Sakornsakolpat, Shabnam Salimi, Steven Salzberg, Kevin Sandow, Vijay Sankaran, Ellen Schmidt, Karen Schwander, David Schwartz, Frank Sciurba, Christine Seidman, Vivien Sheehan, Amol Shetty, Aniket Shetty, Wayne Hui-Heng Sheu, M. Benjamin Shoemaker, Brian Silver, Edwin Silverman, Jennifer Smith, Josh Smith, Nicholas Smith, Tanja Smith, Sylvia Smoller, Beverly Snively, Tamar Sofer, Nona Sotoodehnia, Adrienne Stilp, Elizabeth Streeten, Yun Ju Sung, Jody Sylvia, Adam Szpiro, Carole Sztalryd, Daniel Taliun, Hua Tang, Margaret Taub, Kent Taylor, Simeon Taylor, Marilyn Telen, Timothy A. Thornton, Lesley Tinker, David Tirschwell, Hemant Tiwari, Russell Tracy, Michael Tsai, Dhananjay Vaidya, Peter VandeHaar, Scott Vrieze, Tarik Walker, Robert Wallace, Avram Walts, Emily Wan, Fei Fei Wang, Karol Watson, Daniel E. Weeks, Bruce Weir, Scott Weiss, Lu-Chen Weng, Cristen Willer, Kayleen Williams, L. Keoki Williams, Carla Wilson, Quenna Wong, Huichun Xu, Lisa Yanek, Ivana Yang, Rongze Yang, Norann Zaghloul, Yingze Zhang, Snow Xueyan Zhao, Xiuwen Zheng, Degui Zhi, Xiang Zhou, Michael Zody, Sebastian Zoellner

**Affiliations:** 1 Center for Genomic Medicine and Cardiovascular Research Center, Massachusetts General Hospital, Boston, MA 02114 USA; 2 Department of Medicine, Harvard Medical School, Boston, MA 02115 USA; 3 Broad Institute of Harvard & MIT, Cambridge, MA 02142 USA; 4 Department of Biostatistics, Boston University School of Public Health, Boston, MA 02118 USA; 5 Yale School of Medicine, New Haven, CT 06510 USA; 6 Department of Computational Biology & Bioinformatics, Yale University, New Haven, CT 06520 USA; 7 School of Medicine, University of Maryland, Baltimore, MD 21201 USA; 8 Analytic and Translational Genetics Unit, Massachusetts General Hospital, Boston, MA 02114 USA; 9 Department of Computational Medicine and Bioinformatics, School of Public Health, University of Michigan, Ann Arbor, MI 48109 USA; 10 Society of Fellows, Harvard University, Cambridge, MA 02138 USA; 11 Department of Biological Chemistry, University of California, Los Angeles, Los Angeles, CA 90095 USA; 12 Institute for Molecular Medicine Finland, Helsinki, 00290 Finland; 13 Estonian Genome Center, University of Tartu, Tartu, 51010 Estonia; 14 Center for Public Health Genomics, University of Virginia, Charlottesville, VA 22908 USA; 15 Department of Biostatistics, University of Washington, Seattle, WA 98195 USA; 16 Department of Medicine, University of Mississippi Medical Center, Jackson, MS 39216 USA; 17 Sections of Preventive Medicine and Epidemiology and Cardiology, Department of Medicine, Boston University School of Medicine, Boston, MA 02118 USA; 18 Department of Epidemiology, Boston University School of Public Health, Boston, MA 02118 USA; 19 Framingham Heart Study, Framingham, MA 01702 USA; 20 Computer Science and Artificial Intelligence Lab (CSAIL), Massachusetts Institute of Technology, Cambridge, MA 02139 USA; 21 Department of Biostatistics, School of Public Health, University of Michigan, Ann Arbor, MI 48109 USA; 22 Department of Physiology and Biophysics, University of Mississippi Medical Center, Jackson, MS 39216 USA; 23 Institute for Translational Genomics and Population Sciences, LABioMed and Departments of Pediatrics and Medicine, Harbor-UCLA Medical Center, Torrance, CA 90502 USA; 24 Departments of Human Genetics, Internal Medicine, and Computational Medicine and Bioinformatics, University of Michigan, Ann Arbor, MI 48109 USA; 25 New York Genome Center, New York, NY 10013 USA; 26 Massachusetts General Hospital, Boston, MA 02114 USA; 27 Wake Forest Baptist Health, Winston-Salem, NC 27157 USA; 28 Children’s Hospital of Philadelphia, Philadelphia, PA 19104 USA; 29 University of Pennsylvania, Philadelphia, PA 19104 USA; 30 Emory University, Atlanta, GA 30322 USA; 31 University of Maryland School of Medicine, Baltimore, MD 21201 USA; 32 University of Washington, Seattle, WA 98195 USA; 33 University of Mississippi, Jackson, MS 38677 USA; 34 National Institutes of Health, Bethesda, MD 20892 USA; 35 Johns Hopkins University, Baltimore, MD 21218 USA; 36 University of Kentucky, Lexington, KY 40506 USA; 37 Duke University, Durham, NC 27708 USA; 38 University of Alabama, Birmingham, AL 35487 USA; 39 Stanford University, Stanford, CA 94305 USA; 40 University of Wisconsin Milwaukee, Milwaukee, WI 53211 USA; 41 Cleveland Clinic, Cleveland, OH 44195 USA; 42 University of Colorado at Denver, Denver, CO 80204 USA; 43 Columbia University, New York, NY 10027 USA; 44 Boston University, Boston, MA 02215 USA; 45 Fundação de Hematologia e Hemoterapia de Pernambuco - Hemope, Recife, 52011-000 Brazil; 46 University of Michigan, Ann Arbor, MI 48109 USA; 47 University of Texas Rio Grande Valley School of Medicine, Brownsville, TX 78520 USA; 48 University of Texas Health, Houston, TX 77225 USA; 49 National Jewish Health, Denver, CO 80206 USA; 50 Medical College of Wisconsin, Milwaukee, WI 53226 USA; 51 University of California, San Francisco, San Francisco, CA 94143 USA; 52 Women’s Health Initiative, Seattle, WA 98109 USA; 53 University of California, Los Angeles, Los Angeles, CA 90095 USA; 54 Brigham & Women’s Hospital, Boston, MA 02115 USA; 55 University of Virginia, Charlottesville, VA 22903 USA; 56 Los Angeles Biomedical Research Institute, Los Angeles, CA 90502 USA; 57 The Broad Institute, Cambridge, MA 02142 USA; 58 National Taiwan University, 10617 Taipei, Taiwan; 59 University of Vermont, Burlington, VT 05405 USA; 60 Blood Systems Research Institute UCSF, San Francisco, CA 94118 USA; 61 University of Illinois at Chicago, Chicago, IL 60607 USA; 62 Mayo Clinic, Rochester, MN 55905 USA; 63 Vanderbilt University, Nashville, TN 37235 USA; 64 University of Cincinnati, Cincinnati, OH 45220 USA; 65 Icahn School of Medicine at Mount Sinai, New York, 10029 NY USA; 66 University of North Carolina, Chapel Hill, NC 27599 USA; 67 University of Texas Rio Grande Valley School of Medicine, Edinburg, TX 78539 USA; 68 Washington University in St Louis, St Louis, MO 63130 USA; 69 Brown University, Providence, RI 02912 USA; 70 Fred Hutchinson Cancer Research Center, Seattle, WA 98109 USA; 71 University of Pittsburgh, Pittsburgh, PA 15260 USA; 72 Yale University, New Haven, CT 06520 USA; 73 University of Texas Rio Grande Valley School of Medicine, San Antonio, TX 78229 USA; 74 Tulane University, New Orleans, LA 70118 USA; 75 University of Iowa, Iowa City, IA 52242 USA; 76 National Health Research Institute Taiwan, 350 Zhunan Township, Taiwan; 77 Blood Works Northwest, Seattle, WA 98105 USA; 78 Taichung Veterans General Hospital Taiwan, 407 Taichung City, Taiwan; 79 Ohio State University Wexner Medical Center, Columbus, OH 43210 USA; 80 Blood Works Northwest, Seattle, WA 98106 USA; 81 NIH National Heart, Lung, and Blood Institute, Bethesda, MD 20892 USA; 82 Albert Einstein College of Medicine, New York, NY 10461 USA; 83 Blood Works Northwest, Seattle, WA 98104 USA; 84 Loyola University, Maywood, IL 60153 USA; 85 Harvard School of Public Health, Boston, MA 02115 USA; 86 George Washington University, Washington, DC 20052 USA; 87 Harvard University, Cambridge, MA 02138 USA; 88 University of Arizona, Tucson, AZ 85721 USA; 89 Howard University, Washington, DC 20059 USA; 90 University at Buffalo, Buffalo, NY 14260 USA; 91 University of Minnesota, Minneapolis, MN 55455 USA; 92 Northwestern University, Chicago, IL 60208 USA; 93 Blood Works Northwest, Seattle, WA 98107 USA; 94 Harvard Medical School, Boston, MA 02115 USA; 95 Baylor College of Medicine, Houston, TX 77030 USA; 96 UMass Memorial Medical Center, Worcester, MA 01655 USA; 97 University of Colorado at Boulder, Boulder, CO 80309 USA; 98 Henry Ford Health System, Detroit, MI 48202 USA

## Abstract

Large-scale deep-coverage whole-genome sequencing (WGS) is now feasible and offers potential advantages for locus discovery. We perform WGS in 16,324 participants from four ancestries at mean depth >29X and analyze genotypes with four quantitative traits—plasma total cholesterol, low-density lipoprotein cholesterol (LDL-C), high-density lipoprotein cholesterol, and triglycerides. Common variant association yields known loci except for few variants previously poorly imputed. Rare coding variant association yields known Mendelian dyslipidemia genes but rare non-coding variant association detects no signals. A high 2M-SNP LDL-C polygenic score (top 5th percentile) confers similar effect size to a monogenic mutation (~30 mg/dl higher for each); however, among those with severe hypercholesterolemia, 23% have a high polygenic score and only 2% carry a monogenic mutation. At these sample sizes and for these phenotypes, the incremental value of WGS for discovery is limited but WGS permits simultaneous assessment of monogenic and polygenic models to severe hypercholesterolemia.

## Abstract (web summary)

Common genetic variants associated with plasma lipids have been extensively studied for a better understanding of common diseases. Here, the authors use whole-genome sequencing of 16,324 individuals to analyze rare variant associations and to determine their monogenic and polygenic contribution to lipid traits.

## Introduction

Plasma lipids, including total cholesterol, low-density lipoprotein cholesterol (LDL-C), high-density lipoprotein cholesterol (HDL-C), and triglycerides, are heritable risk factors for atherosclerotic cardiovascular disease^1,2^. Understanding the inherited basis for plasma lipid levels has led to new treatments and to tests to identify individuals at risk for disease. Advances in technologies to characterize DNA sequence variants (i.e., Sanger sequencing, genotyping arrays, exome sequencing) have progressively allowed us to solve monogenic forms of dyslipidemia and to uncover common DNA sequence variants as well as rare mutations that contribute to plasma lipid levels in the population. However, due to the inherent limitations of genotyping arrays and exome sequencing, the non-coding regions of the genome remains incompletely characterized, particularly for rare mutations. In addition, the relative contribution of common DNA sequence variants and rare coding mutations to extreme lipid values in the population has not been delineated.

It is now possible to directly enumerate the whole-genome sequences of a large number of individuals. When performed at sufficient depth of coverage (>20-fold coverage per base), whole-genome sequencing (WGS) can detect single nucleotide polymorphisms (SNPs), insertions, and deletions across the allele frequency spectrum in both non-coding and coding regions. These advances allow us to test the incremental value of WGS as a tool for locus discovery and also develop a framework to understand why a specific individual might have an extreme lipid value. Toward these two goals, we studied the whole-genome sequences in 16,324 participants of European, African, East Asian, and Hispanic ancestries with available plasma lipids phenotypes.

In common variant association analyses, we replicate prior loci but detect newly associated variants not previously detected by prior genome-wide genotyping arrays or imputation. Analyses of rare coding variants yield known Mendelian dyslipidemia genes. Four approaches for analyzing rare non-coding variant associations do not detect any signals. WGS analysis of severe hypercholesterolemia shows a ten-fold enrichment of a high polygenic LDL-C score versus monogenic mutation for severe hypercholesterolemia. While the incremental value for WGS for locus discovery currently is limited largely due to relatively smaller sample sizes, WGS markedly improves the diagnostic yield of severe hypercholesterolemia through simultaneous assessment of monogenic and polygenic models.

## Results

### Deep-coverage WGS of 16,324 participants

**Fig. 1:**
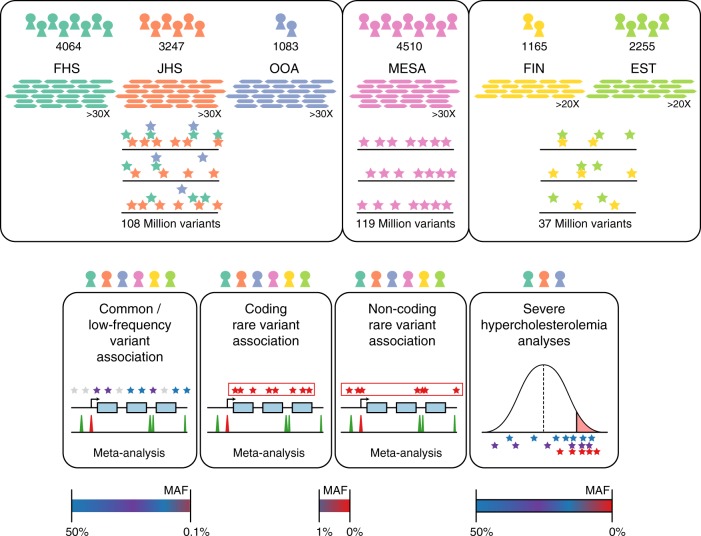
Schematic of genomic variant discovery and analyses. Variants were jointly discovered in three distinct sets: (1) FHS, JHS, and OOA; (2) MESA; and (3) EST and FIN. Cohorts included in analyses are denoted by color-coded icons. Allele frequency spaces assessed are indicated for analyses. EST Estonia, FHS Framingham Heart Study, FIN Finland, JHS Jackson Heart Study, MESA Multi-Ethnic Study of Atherosclerosis, OOA Old Order Amish

Participants of the Framingham Heart Study (FHS), Old Order Amish (OOA), Jackson Heart Study (JHS), Multi-Ethnic Study of Atherosclerosis (MESA), FINRISK Study (FIN), and Estonian Biobank (EST) underwent WGS (Fig. 1). Following quality control (Supplementary Table 1), 16,324 participants with plasma lipids available were included in the analysis (Supplementary Table 2). The mean (standard deviation (SD)) age was 51 (15) years and 8669 (53%) were women. About 5911 (36%) of the participants were of non-European ancestry (Supplementary Table 2, Supplementary Fig. 1a-c. The proportion of individuals on lipid-lowering medications was low (9%).

WGS target coverage was >30X for FHS, OOA, JHS, and MESA (as a part of the NIH/NHLBI Trans-Omics for Precision Medicine (TOPMed) research program) and was >20X for EST and FIN (Supplementary Fig. 2). The mean (SD) attained coverage for >30X target samples was 37.1(5.4)X and for >20X target was 29.8(5.4)*X*.

**Fig. 2:**
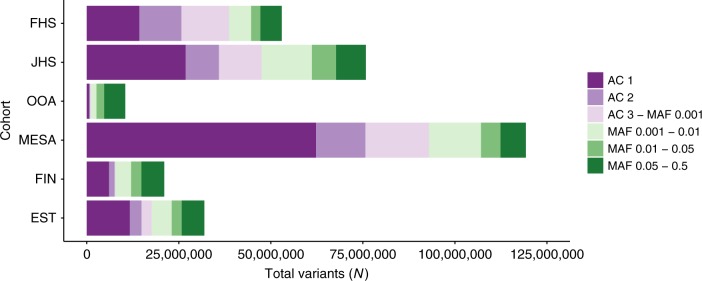
Deep-coverage WGS identifies genomic variation across the allelic spectrum. Variant counts by allele count/frequency bin within each of the cohorts. Singletons (“AC 1”) and doubletons (“AC 2”) are separately distinguished from allele frequency bins within each cohort. Variants were jointly discovered in three distinct sets: (1) FHS, JHS, and OOA; (2) MESA; and (3) EST and FIN. AC allele count, EST Estonia, FHS Framingham Heart Study, FIN Finland, JHS Jackson Heart Study, MAF minor allele frequency, MESA Multi-Ethnic Study of Atherosclerosis, OOA Old Order Amish

After performing quality control, a total of 189 million unique variants were discovered across all datasets. Total variant count characteristics varied by cohort due to sample sizes, relatedness, ethnicity, and population history (Fig. 2). As expected, the MESA cohort, of largely unrelated individuals of four diverse ethnicities, had the most variants per individual while the OOA cohort, a founder population of European ancestry, had the fewest variants per individual (Supplementary Table 3). The median number of variants, or sites with alleles differing from the hg19 reference genome, per individual was 3,391,000, of which on average 4878 were observed in only a single individual.

### Common plus low-frequency variant association study

We first analyzed common and low-frequency variants, i.e., those that occur with enough minor alleles to provide robust individual association test statistics. We considered variants that had a minor allele frequency (MAF) >0.1% within at least one of the three WGS variant callsets (minor allele count >16 for the FHS/OOA/JHS callset, >9 for MESA, or >6 for FIN and EST) (Fig. 1). Association for these variants was estimated within each callset with each of the four plasma lipids levels, and then meta-analyzed using the inverse-variance method. Overall, 32,086,348 variants were included in this analysis. The test statistics were well controlled (Supplementary Table 4 and Supplementary Fig. 3a-d). We used a conventional statistical threshold for genome-wide significance (*α* = 5 × 10^−8^)^3^ (Supplementary Fig. 3e-h). Using this cutoff, 592, 697, 447, and 522 variants were associated with total cholesterol, LDL-C, HDL-C, and triglycerides, respectively (Supplementary Table 5). These variants were distributed at 10, 7, 13, and 9 loci previously associated with total cholesterol, LDL-C, HDL-C, and triglycerides, respectively, and five at putative novel lipid loci (Supplementary Table 6)^4–7^. Of the variants at known loci, 12 (38.7%) were lead variants in prior associations, eight (25.8%) new lead variants were in high linkage disequilibrium (LD) (*r*^2^ > 0.8) with prior lead variants, and the remaining 11 (35.5%) new lead variants were in low LD (*r*^2^ < 0.2) with prior lead variants.

At a conventional *α* threshold of 5 × 10^−8^, we discovered five associations at putative novel lipid loci (Supplementary Table 6). For example, rs3215707 (MAF 2.0%), a 1-bp deletion at 9p24.1, was associated with HDL-C (+3.3 mg/dl, *P* = 1.3 × 10^−8^). rs3215707 occurs within an intron of *PLGRKT* and overlies active promoter and strong enhancer histone modification signals for HepG2 cells (Supplementary Fig. 4). The deletion is not in LD with any known SNPs and thus the association was not detectable by prior genome-wide association analyses. Within each callset, estimated effects were consistent (heterogeneity *P* = 0.53) and all demonstrated at least nominal association (*P* < 0.05) (Supplementary Table 7). We sought further replication for rs3215707 from additional independent samples. We interrogated 233 individuals from families with dyslipidemia and enriched for premature coronary heart disease who were whole-genome sequenced within the EUFAM study^8^. Using a mixed model, carriers (MAF 5.1%) were associated with a 5.6 mg/dl greater HDL cholesterol (*P* = 0.03).

We performed iterative conditional analyses to identify distinct independent associations among 16 loci reaching *P* < 5 × 10^−8^ for LDL-C, HDL-C, and triglycerides in the FHS/OOA/JHS (TOPMed Phase I) variant call file (VCF). While only four (25%) loci displayed evidence of allelic heterogeneity at *P* < 5 × 10^−8^, 13 (81.3%) had at least moderate evidence (*P* < 1 × 10^−4^) of allelic heterogeneity across the different ethnic groups available (Supplementary Table 8). Through conditional analyses for LDL-C, we identified a low-frequency haplotype specific to African Americans (MAF 0.1% FHS, 0% OOA, 1.0% JHS,) including variants in LD (*r*^2^ > 0.8) at a transcriptional transition region within the first intron of *LDLR* (rs17242843), *LDLR* promoter (rs17249141), and enhancer 4 kb upstream from the *LDLR* transcription start site (TSS) (rs114197570) (Supplementary Fig. 5, Supplementary Fig. 6). Presence of these variants resulted in a 28 mg/dl lowering of LDL-C (*P* = 2 × 10^−11^), suggesting increased expression of *LDLR* for carriers of the minor allele (Supplementary Fig. 7).

### Rare variant association study of coding variants

To improve the power of detecting rare variant associations, we aggregated putative disruptive rare variants in coding sequences of each gene and tested the quantitative trait distribution among carriers of a set versus non-carriers^9^. We aggregated coding sequence variants within each gene that were predicted to lead to loss of function (e.g., nonsense, canonical splice-site, or frameshift) or annotated as “disruptive” by the ensemble MetaSVM^10^ in silico approach. The median combined MAF per gene was 0.25% [interquartile range 0.090–0.69%] (Supplementary Fig. 8). To account for known bidirectional effects of disruptive mutations in some Mendelian dyslipidemia genes, we accordingly used a mixed model Sequence Kernel Association Test (SKAT)^11,12^. Six genes associated with lipids at an exome-wide level (*α* = 0.05/~20,000 protein-coding genes = 2.5 × 10^−6^) (*LDLR*, *APOB*, *PCSK9*, and *APOE* for LDL-C, *LCAT* for HDL-C, and *APOC3* for triglycerides). Each has been previously established as a cause of Mendelian forms of dyslipidemia (Supplementary Table 9).

### Rare variant association study of non-coding variants

**Fig. 3:**
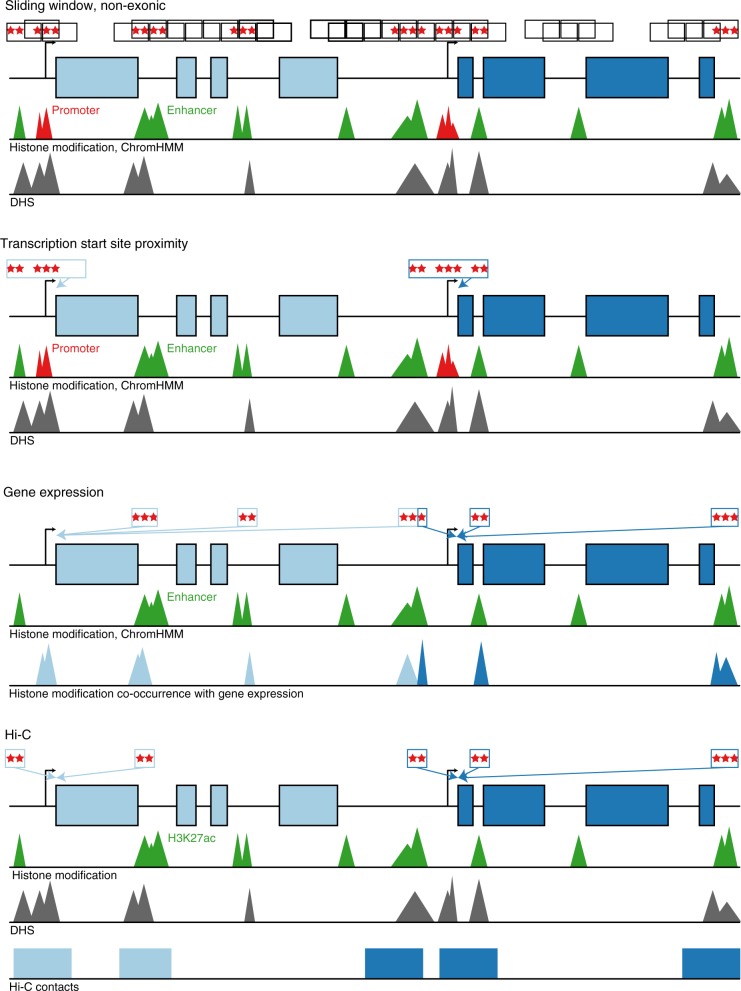
Schematic of non-coding rare variant analyses. Four grouping schematics of rare non-coding variants (MAF <1%). (1) The sliding window approach tiles across the genome at fixed widths, only including variants overlying annotations consistent with enhancers, promoters, and DHS in non-exonic regions. All other approaches attempt to map non-coding putative functional genomic regions with discrete genes as the analytical unit. Overall, they are based on: (2) promoter, enhancer, and DHS annotations near a gene’s transcription start site, (3) co-occurrence of enhancer and DHS annotations with HepG2 gene expression, and (4) H3K27ac marks within Hi-C contact regions mapped to genes. DHS DNase hypersensitivity site, MAF minor allele frequency

Next, we sought to determine whether rare variants in non-coding regions associate with plasma lipids. We used four approaches to aggregate rare, non-coding variants. (Fig. 3). First, we aggregated variants within “sliding windows” of 3 kb in length^13,14^. Second, we connected a non-coding variant to a gene if it resided in a segment annotated as an enhancer (and within 20 kb of a gene) or a region annotated as a promoter (and within 5 kb of the TSS of a gene). Third, using gene expression information, we connected a non-coding variant to a gene if it resided in a region annotated as an enhancer. Finally, we connected a non-coding variant to a gene based on a model which predicted gene-enhancer pairs using a chromatin-state model, including both HK27ac and Hi-C contact data, that we previously described^15^. Regulatory annotations were derived from the ENCODE and NIH Roadmap projects for two cell types—HepG2 and adipose nuclei—relevant to lipoprotein metabolism. For these analyses, we considered a *P* < 0.05 / 254,032 groups = 2.0 × 10^−7^ as significant (Supplementary Table 10, Supplementary Table 11).

Using the sliding window approach to non-coding burden tests, we observed suggestive associations for 3 kb windows at the *CETP* (start chr16:56667000) locus (minimum *P* = 4 × 10^−6^) and at the *APOA1-C3-A4-A5* (start chr11:117094500) locus (minimum *P* = 8 × 10^−6^) with HDL-C. A total of 17.6% of non-coding sliding windows occurring within 1 Mb of known lead lipid variants were at least nominally (*P* < 0.05) associated with lipids versus 4.4% in other regions of the genome across all traits (*P* difference = 8 × 10^−272^).

An aggregation of rare non-coding variants at only two genes—*LDLR* and *APOE*—were associated with LDL-C and total cholesterol (*P* < 2 × 10^−7^) (Supplementary Fig. 9) (Supplementary Table 12). The strongest *LDLR* signal (*P* = 9.7 × 10^−11^) was seen for an analysis that connected enhancers and promoters to a gene based on physical proximity (approach #2 above). Closer inspection of the specific variants shows that this signal is driven by the low-frequency haplotype specific to African Americans also detected with single variant association (Supplementary Fig. 10) (Supplementary Table 12). The strongest *APOE* signal (*P* = 8.1 × 10^−26^) was observed in the model connecting enhancers to a gene by eQTLs for gene expression (approach #3 above). However, accounting for the strongest common variant association at the locus (rs7412, the *APOE* ε2 isoform allele), this signal attenuates to non-significance (*P* *=* 1.8 × 10^−2^), suggesting that the non-coding variants are driven by LD of the *APOE* ε2 isoform. Beyond these two results, we found no additional signals for a burden of non-coding variants.

### Contribution of mono- and polygenic models to extreme LDL-C

With the availability of sequence in both coding and non-coding regions in the same samples, we estimated the simultaneous contribution of monogenic and polygenic determinants to extreme LDL-C in a population-based sample of European (EA) and African (AA) ancestry. We defined “extreme” as the top or bottom 5th ancestry-specific percentile of LDL-C. Analyses were conducted in FHS and MESA-EA subjects (extreme cutoff as LDL-C >183 mg/dl or LDL-C <72.9 mg/dl) and JHS and MESA-AA subjects (extreme cutoff as LDL-C >198.6 mg/dl or LDL-C <71 mg/dl), separately.

Among participants with extremely high LDL-C, we searched for mutations in any of six Mendelian genes previously implicated as causing elevated LDL-C (*LDLR*, *APOB*, *PCSK9*, *ABCG5*, *ABCG8*, and *LDLRAP1*) (Supplementary Table 13).

To determine polygenic contribution, we implemented a systematic approach to derive, test, and validate a new “genome-wide” polygenic score for LDL-C using mutually independent datasets. A polygenic score provides a quantitative assessment of the cumulative risk associated with multiple common risk alleles for each individual.

We derived polygenic scores by three approaches: (1) only inclusion of genome-wide significant variants (*P* < 5 × 10^−8^ in separate discovery)^7^, (2) *r*^2^ and *P* value thresholds to restrict variants without rescaling weights, and (3) entire summary results of 2M variants (LDPred) with rescaled weights based on *r*^2^ and *P* values^16^. We derived polygenic scores based on the association statistics of all available common (MAF ≥ 0.01) SNPs with LDL-C, as determined by our previously published genome-wide association study^7^.

As a baseline, we generated an additional polygenic score restricted to lead variants (*P* *<* 5 × 10^−8^) at distinct genomic loci, weighted by discovery estimated effects (“restricted score”). Second, we applied various *r*^2^ and *P* value thresholds to the previously published results. Finally, we used the LDPred computational algorithm which constructs genome-wide polygenic scores across full summary statistics^16^. Prior simulations have suggested that approaches additionally including variants with sub-genome-wide significance may improve the predictive capability of polygenic risk scores^17^. To include such variants, LDPred re-weights corresponding per-variant weights from our prior genome-wide association study^7^ based on LD, discovery *P* values, and a range of estimated causal fraction (e.g., non-zero effect sizes) markers. The correlation between the variants was assessed using the European reference population from the 1000 Genomes study^17^. The best score was determined based on maximal model fit (*R*^2^) from a linear regression models in a health-care biobank of 25,534 unrelated individuals (Nord-Trøndelag Health Study, HUNT)^18^ (Supplementary Table 14).

For LDL-C, a genome-wide polygenic score incorporating 2 million SNPs with LDpred provided the best model fit (Supplementary Table 15). Compared to a restricted score of 59 SNPs independently significant associated with LDL-C, a relative increase of 21.6% of LDL-C variance was explained by the expanded 2M-SNP score (*r*^2^_restricted_ = 0.245 vs. *r*^2^_expanded_ = 0.298). We applied this polygenic score separately within the WGS samples in FHS, JHS, and MESA. We labeled individuals as having a high polygenic score if they fell in the top 5th percentile of race-specific score distributions (Tables 1 and 2).

**Table 1:** Effect of monogenic mutation or polygenic score on odds for extremely high or low LDL-C

Extremely high LDL-C										
Ancestry	*N*_total_	*N*_extreme_	Monogenic carrier (*N*_extreme_)	High polygenic score (*N*_extreme_)	Monogenic carrier OR (95% CI)	Monogenic carrier *P*-value	Monogenic carrier PAF	High polygenic score OR (95% CI)	High polygenic score *P*-value	Top 5th percentile of polygenic score PAF
EA	5910	284	5	64	10.92 (3.71, 32.14)	1.4 × 10^−5^	1.60	7.65 (5.56, 10.52)	5.7 × 10^−36^	19.6
AA	4380	217	7	29	7.43 (3.01, 18.35)	1.4 × 10^−5^	2.79	3.2 (2.1, 4.89)	6.7 × 10^−8^	9.2
**Extremely Low LDL-C**										
**Ancestry**	***N***_**total**_	***N***_**extreme**_	**Monogenic carrier** (***N***_**extreme**_)	**Low polygenic score (** ***N***_**extreme**_ **)**	**Monogenic carrier OR (95% CI)**	**Monogenic carrier** ***P*** **-value**	**Monogenic carrier PAF**	**Low polygenic score OR (95% CI)**	**Low polygenic score** ***P*** **-value**	**Bottom 5th percentile of polygenic score PAF**
EA	5910	286	6	82	21.73 (6.2, 76.15)	1.5 × 10^−6^	2.00	10.38 (7.69, 14.02)	1.5 × 10^−52^	25.9
AA	4380	218	11	32	13.83 (6.25, 30.62)	9.4 × 10^−11^	4.68	3.7 (2.46, 5.58)	3.9 × 10^−10^	10.7

Values are represented as OR [95% CI] for association with given trait. (b). Effect of monogenic mutation or polygenic score on LDL-C in mg/dl. Values are represented as beta [95% CI] in mg/dl for LDL-C. Multi-variable associations were performed with sex + age + age^2^ (effects not listed) with monogenic carrier status + high polygenic score using logistic regression. Polygenic risk score was derived from 2 million variants using LDpred. High polygenic score was defined as membership in the top 5th percentile of the ancestry-specific score distribution. AA, African American; EA, European American; SE, standard error.

**Table 2:** Effect of monogenic mutation or polygenic score on LDL-C in mg/dl

Monogenic mutation or high polygenic score									
Ancestry	*N*_total_	Monogenic carrier (*N*)	High polygenic score (*N*)	Monogenic carrier *β* (mg/dl)	Monogenic carrier SE	Monogenic carrier *P*-value	Highpolygenic score *β* (mg/dl)	High polygenic score SE	High polygenic score *P*-value
EA	5910	18	297	29.98	8.07	2.1 × 10^−4^	33.07	2.05	1.7 × 10^−57^
AA	4380	25	220	41.05	7.93	2.3 × 10^−7^	16.96	2.74	6.4 × 10^−10^
**Monogenic mutation or low polygenic score**									
**Ancestry**	***N***_**total**_	**Monogenic carrier (** ***N*** **)**	**Low polygenic score (** ***N*** **)**	**Monogenic carrier** ***β*** **(mg/dl)**	**Monogenic carrier SE**	**Monogenic carrier** ***P*** **-value**	**Low polygenic score** ***β*** **(mg/dl)**	**Low polygenic score SE**	**Low polygenic score** ***P*** **-value**
EA	5910	12	297	−47.25	9.55	7.7 × 10^−7^	−35.00	2.00	7.9 × 10^−67^
AA	4380	28	220	−41.41	7.47	3.1 × 10^−8^	−20.41	2.74	1.1 × 10^−13^

Values are represented as beta [95% CI] in mg/dl for LDL-C. Multi-variable associations were performed with sex + age + age^2^ (effects not listed) with monogenic carrier status + high polygenic score using linear regression. Polygenic risk score was derived from 2 million variants using LDpred. High polygenic score was defined as membership in the top 5th percentile of the ancestry-specific score distribution. AA, African American; EA, European American; SE, standard error.

Among EA participants, a monogenic mutation was associated with an odds ratio of 10.92 (95% CI 3.71(32.14) for extremely high LDL-C, whereas a high polygenic score associated with an odds ratio of 7.65 (95% CI 5.56–10.52). In EA individuals, those who carried a monogenic mutation had 30 mg/dl higher LDL-C (when compared with non-carriers; *P* = 2.1 × 10^−4^) and those who had a high polygenic score had 33 mg/dl greater LDL-C (when compared with all others; *P* = 1.7 × 10^−57^). Of the 287 EA participants with extremely high LDL-C, 2% carried a monogenic mutation and 23% had a high polygenic score.

Among AA participants, a monogenic mutation was associated with an odds ratio of 7.43 (95% CI 3.01–18.35) for extremely high LDL-C, whereas a high polygenic score associated with an odds ratio of 3.2 (95% CI 2.1–4.89). In AA individuals, those who carried a monogenic mutation had 41 mg/dl higher LDL-C (when compared with non-carriers; *P* = 2.3 × 10^−7^), greater than that observed among EA individuals, and those who had a high polygenic score had 17 mg/dl greater LDL-C (when compared with all others; *P* = 6.4 × 10^−10^), less than the effect observed among EA individuals. Of the 217 AA participants with extremely high LDL-C, 3% carried a monogenic mutation and 13% had a high polygenic score. Across the full spectrum of LDL-C polygenic score, every SD of the LDL-C polygenic score was associated with 15.5 mg/dl LDL-C among EA (*P* = 4 × 10^−277^) and 8.7 mg/dl LDL-C among AA (*P* = 1 × 10^−47^).

We replicated the association between a high polygenic score and extremely high LDL-C in an independent sample, the ARIC cohort. Among ARIC-EA (*N* = 7755) individuals, a high polygenic score was associated with an odds ratio of 7.35 (95% CI 5.95–9.10; *P* < 2 × 10^−16^) for extremely high LDL-C and 42.8 mg/dl (95% CI 40.0–47.5; *P* < 2 × 10^−16^) higher LDL-C compared with individuals without a high polygenic score. Among ARIC-AA (*N* = 1907) participants, a high polygenic score was associated with an odds ratio of 2.7 (95% CI 1.77–4.09; *P* < 3.3 × 10^−6^) for extremely high LDL-C and a 23.2 mg/dl (95% CI 15.0–31.5; *P* = 3.8 × 10^−8^) higher LDL-C compared with individuals without a high polygenic score.

We analyzed the monogenic and polygenic contribution to extremely low LDL-C in EA and AA participants and found similar patterns where monogenic mutations as well as a polygenic score conferred similar effect sizes (Tables 1 and 2).

## Discussion

We performed WGS in 16,342 ethnically diverse individuals and analyzed the incremental value of WGS for locus discovery for blood lipid levels and for clinical interpretation. We replicated associations for 28 common variant loci previously associated with lipids in much larger genome-wide association analyses. We identified an association for a low frequency 1-bp deletion at 9p24.1 with HDL-C. We replicated burden associations of rare coding mutations at known Mendelian lipid genes. However, we did not detect any burden associations of rare non-coding mutations through four different approaches. Lastly, we developed a genome-wide polygenic score and showed that such a score confers an effect size on LDL-C similar to carrying a monogenic mutation and is present in ten-fold more individuals with severe hypercholesterolemia than monogenic mutations. At these sample sizes and for these phenotypes, the incremental value of WGS as a discovery tool was limited but WGS allowed us to simultaneously assess the contribution of monogenic and polygenic models to severe hypercholesterolemia.

These results permit several conclusions. Using WGS as a discovery tool, the incremental yield of new loci was modest. Current sample sizes for WGS are much smaller compared to genome-wide association and whole exome sequencing studies clearly limiting relative power for detecting associations for common/low-frequency non-coding variants and rare coding variants. Despite genome-wide interrogation of rare variant signals in non-coding space, we identified no burden-of-rare-variant signals using four different aggregation approaches and regulatory annotations from two relevant tissues.

Mutation target size and natural selection pressure are smaller in non-coding regions when compared with coding regions; based on these considerations, power calculations have suggested that sample sizes may need to be considerably larger to identify rare variant burden associations in non-coding regions compared to coding regions^9^. While sample size is an important determinant of power, prioritization of putative causal rare non-coding variants remains a major power limitation. Functional annotations from reference datasets largely prioritize functional sequence and MAF thresholds assist in prioritizing causal variants, but this likely retains a large fraction of benign variants. Genome-wide organism-level functional variant scores^19^ offer the promise of improved prioritization but did not improve associations at *LDLR* and *APOE*. Novel, genome-wide tissue-level functional scores may improve prioritization compared to organism-level scores^20–22^. Assessments of consequence for rare coding mutations in experimental systems has improved associations of lipid-related genes beyond in silico tools^23,24^. Similar systematic approaches for rare non-coding variants in relevant tissues may further improve power.

WGS in diverse populations permits discovery of novel associated variants. Most of the observed lead single variant associations at known loci were previously tagged by lead variants from genome-wide association analyses of largely European ancestry participants. Our trans-ethnic analyses yielded new lead variants at one-third of known lipid loci not previously tagged by prior lead loci. Additionally, variant classes not previously detected by array-genotyping and whole-exome sequencing are associated with lipids. We observed that a 1-bp deletion, not correlated with previously cataloged variants, was associated with HDL cholesterol. These observations indicate that new variants are detected not only by including diverse ethnicities, but also WGS can overcome many limitations of imputation for variant discovery, including application in non-Europeans, variable coverage in genome-wide genotype arrays, and detection of rarer variants.

Of great interest, we observed that the relative contribution of polygenic score to extremely high LDL-C is considerably greater than monogenic mutations. For example, in EA individuals, both high polygenic score and a monogenic mutation confer similar effects (~30 mg/dl higher LDL-C) but a high polygenic score is present in 20% of participants with extremely high LDL-C whereas a monogenic mutation is present in only 2%. In most individuals who carry diagnosis of familial hypercholesterolemia, no monogenic mutation is identified with clinical exome sequencing;^25,26^ for a large fraction of these “mutation-negative” familial hypercholesterolemia, high polygenic scores may be operative. WGS permits the application of simultaneous assessment of monogenic determinants as well as the most optimally performing polygenic score with relative ease.

Our observed monogenic carrier rates for severe hypercholesterolemia (2%) are consistent with observations in other population-based cohorts^26,27^ and health-care-associated biobanks^25^ but lower than for patients with clinical criteria for familial hypercholesterolemia (up to 24%)^27^, particularly those clinically referred for familial hypercholesterolemia genetic testing (up to 50%)^28–31^. As anticipated, this subgroup is also likely to have a greater monogenic relative to polygenic contribution^32,33^.

Important limitations should be considered. First, appropriate definitions of statistical significance for WGS association analyses have not been harmonized in the field. The convention of *α* = 5 × 10^−8^ comes from the assumption of performing 1,000,000 independent tests. Based on our findings and simulations from others^3^, 10^−9^ may be more appropriate for analyses across diverse ethnicities to allele frequency 0.1%. Second, power is somewhat diminished with our rare variant meta-analysis approach to combine *P* values with Fisher’s method. Given known diverse coding mutations within Mendelian genes with bidirectional effects and the inability to assume unidirectional effects within the non-coding space, we employed a SKAT statistical framework. Prior approaches leveraging covariance matrices for SKAT meta-analysis were computationally inefficient for the dataset and multiple grouping strategies^34,35^. Thus, our approach is conservative. Third, the polygenic scores described here were derived from genome-wide association studies performed largely in EA ancestry participants^7^. Because allele frequencies, LD patterns, and effect sizes of common polymorphisms vary by ancestry, the predictive capacity of polygenic score was attenuated in non-European ancestry individuals^36^. Furthermore gene flow between ancestral groups and resultant admixture^37,38^ for an individual further hinders accuracy of polygenic risk scores derived from distinct populations for application at the individual level^39^. This is an important limitation for the field that requires efforts to characterize common genomic variation influencing complex traits among non-Europeans and develop locus admixture-aware polygenic risk scoring.

In summary, we present a large-scale WGS analysis of plasma lipids in 16,324 ethnically diverse participants. Common, non-coding variants and rare, coding variants contribute to plasma lipid variation; however, association signals for rare, non-coding mutations were not detectable. Among participants with severe hypercholesterolemia, a high polygenic score was present in ten-fold more individuals than a monogenic mutation.

## Methods

### Study participants

Study participants were from the FHS (*N* = 4064), JHS (*N* = 3247), OOA (*N* = 1083), MESA (*N* = 4510), FIN (*N* = 1165), and the EST (*N* = 2255). Each study was previously approved by respective institutional review boards (IRBs), including for the generation of WGS data and association with phenotypes. All participants provided written consent. The analyses of WGS data with plasma lipids was approved by the Massachusetts General Hospital IRB (MGH IRB# 2016P001308). Please refer to Supplementary Note 1 for study participant details.

### WGS, variant calling, and genotyping

Sequencing was performed at one of the four sequencing centers, with all members within a cohort sequenced at the same center. For the TOPMED phase 1 data, 4148 FHS individuals and 1095 OOA individuals were sequenced at the Broad Institute of Harvard and MIT (Cambridge, MA), while 3266 JHS individuals were sequenced at University of Washington Northwest Genomics Center (Seattle, WA). About 4601 MESA individuals were additionally sequenced at the Broad Institute of Harvard and MIT as part of TOPMED Phase 2. About 1180 Finnish FINRISK individuals and 2281 Estonian Biobank participants were sequenced at the Broad Institute of Harvard and MIT (Cambridge, MA). Three separate callsets were utilized due to timeline of availability as well as data use restrictions.

TOPMED phase 1 BAM files provided by the sequencing centers were harmonized by the TOPMed Informatics Research Center (IRC) before joint variant discovery and genotype calling across studies. In brief, sequence data were received from each sequencing center in the form of bam files mapped to the 1000 Genomes hs37d5 build 37 decoy reference sequence. Processing was coordinated and managed by the “GotCloud” processing pipeline^40^.

The two sequence quality criteria used in order to pass sequence data on for joint variant discovery and genotyping are: estimated DNA sample contamination below 3%, and fraction of the genome covered at least 10 × 95% or above. DNA sample contamination was estimated from the sequencing center read mapping using software verifyBamId^41^.

The genotype callsets used for analysis are from “freeze 3a” of the variant calling pipeline performed by the TOPMed IRC (Center for Statistical Genetics, University of Michigan, Hyun Min Kang, Tom Blackwell, and Goncalo Abecasis). The software tools used in this version of the pipeline are available in the following repository: https://github.com/statgen/topmed_freeze3_calling. Variant detection (SNPs and indels) from each sequenced (and aligned) genome is performed by vt discover2 software tool^42^. The variant calling software tools are under active development; updated versions can be accessed at http://github.com/atks/vt or http://github.com/hyunminkang/apigenome.

WGS for MESA, FINRISK, and the Estonian Biobank was performed using the Illumina HiSeqX platform at the Broad Institute of Harvard and MIT (Cambridge, MA). DNA samples are informatically received into the Genomics Platform’s Laboratory Information Management System via a scan of the tube barcodes using a Biosero flatbed scanner. All samples are then weighed on a BioMicro Lab’s XL20 to determine the volume of DNA present in the sample tubes. Following this, the samples are quantified in a process that uses PICO-green fluorescent dye. Once volumes and concentrations are determined, the samples are then handed off to the Sample Retrieval and Storage Team for storage in a locked and monitored −20 °C walk-in freezer.

Libraries were constructed and sequenced on the Illumina HiSeqX with the use of 151-bp paired-end reads for WGS and output was processed by Picard to generate aligned BAM files (to hg19)^43,44^. Samples were tracked by automated LIMS messaging. Samples were fragmented with acoustic shearing and libraries were prepared with a KAPA Biosystems kit. Libraries were normalized to 1.7 nM. Cluster amplification was performed using Illumina cBot and flowcells were sequenced in HiSeq X. Variants (SNPs and indels) were discovered using the Geome Analysis Tookit (GATK) v3 HaplotypeCaller according to Best Practices^45^. Variants from MESA samples were generated in one callset. Finland and Estonia samples were jointly called in a separate callset.

### Whole-genome sequence quality control

The following three approaches were used by the TOPMed Genetic Analysis Center to identify and resolve sample identity issues: (1) concordance between annotated sex and biological sex inferred from the WGS data, (2) concordance between prior SNP array genotypes and WGS-derived genotypes, and (3) comparisons of observed and expected relatedness from pedigrees.

The variant filtering in TOPMed Freeze 3 were performed by (1) first calculating Mendelian consistency scores using known familial relatedness and duplicates and (2) training SVM classifier between the known variant sites (positive labels) and the Mendelian inconsistent variants (negative labels). Two additional hard filters were applied: (1) Excess heterozygosity filter (EXHET), if the Hardy–Weinberg disequilbrium *P*-value was less than 1 × 10^−6^ in the direction of excess heterozygosity. An additional ~3900 variants were filtered out by this filter, and (2) Mendelian discordance filter (DISC), with 3 or more Mendelian inconsistencies or duplicate discordances observed from the samples. An additional ~370,000 variants were filtered out by this filter. Functional annotation for each variant was provided in the INFO field using snpEff 4.1 with a GRCh37.75 database^46^. Analysis used hard-call genotypes, without genotype likelihoods. Genotypes with a depth <10 were excluded.

Additional measures for quality control of TOPMed Phase I Freeze 3 and quality control for MESA, Finland, and Estonia were performed using the Hail software package (https://hail.is)^47^. Samples were filtered by contamination (>3.0% for all, except >5.0% for Finland and Estonia), chimeras >5%, GC dropout >4, raw coverage (<30X for all, except <19X for Finland and Estonia), indeterminant genotypic sex or genotypic/phenotypic sex mismatch.

Variants for MESA, Finland, and Estonia were initially filtered by GATK Variant Quality Score Recalibration. Additionally, genotypes with GQ <20, DP < 10 or >200, and poor allele balance (homozygous with <0.90 supportive reads or heterozygous with <0.20 supportive reads) were removed. And variants within low complexity regions were removed across all samples^48^. Variants with >5% missing calls, quality by depth <2 (SNPs) or <3 (indels), InbreedingCoeff <−0.3, and pHWE <1 × 10^−9^ (within each cohort) were filtered out.

### Annotation

Variants were annotated with Hail using annotations from Ensembl’s Variant Effect Predictor^49^ for protein-coding annotations and Reg2Map HoneyBadger2-intersect for regulatory annotations at DNase I regions –log_10_(*P*) ≥10 (https://personal.broadinstitute.org/meuleman/reg2map/HoneyBadger2-intersect_release/).

### Traits

Conventionally measured plasma lipids, including total cholesterol, LDL-C, HDL-C, and triglycerides, were included for analysis. LDL-C was either calculated by the Friedewald equation when triglycerides were <400 mg/dl or directly measured. Given the average effect of statins, when statins were present, total cholesterol was adjusted by dividing by 0.8 and LDL-C by dividing by 0.7, as previously done^50^. Triglycerides were natural log transformed for analysis. Phenotypes were harmonized by each cohort and deposited into the dbGaP TOPMed Exchange Area.

### Common plus low-frequency variant association analysis

Single variant analysis was performed in EPACTS (https://genome.sph.umich.edu/wiki/EPACTS) with Efficient Mixed-Model Association eXpedited (EMMAX) for associating each variant site with each lipid trait as a continuous measure within each jointly called VCF^11^. Empiric kinship matrices were first generated for each VCF (“make-kin”) using default parameters. Next, association analyses (“single”) were performed adjusting for age, age^2^, sex, cohort, self-reported ethnicity (for MESA), and an empirically derived kinship matrix to account for both familial and more distant relatedness within each VCF. For the TOPMed Phase I VCF, which included OOA, LDL-C and total cholesterol analyses were also adjusted for *APOB* p.R3527Q and triglycerides and HDL-C analyses were also adjusted for *APOC3* p.R19Ter. To ensure robust results, we only performed single variant analysis for variants with a MAF >0.1%. Variants were meta-analyzed across all three VCFs using METAL (https://genome.sph.umich.edu/wiki/METAL)^51^. Summary statistics only for variants with MAF >0.1% for the given VCF were included in the meta-analysis. Statistical significance *α* of 5 × 10^−8^ was used for these analyses.

For loci with at least one variant with *P* < 5 × 10^−8^ within the TOPMed Phase I VCF, iterative conditional association analysis was performed. Iterative conditioning was performed until *P* > 1 × 10^−4^ was attained.

### Rare variant association analyses

We first identified rare (MAF <1%) mutations for each VCF within the coding sequences. After Variant Effect Predictor^49^ annotation, we identified loss-of-function (e.g., nonsense, canonical splice-site, and frameshift) and disruptive missense (by MetaSVM^10^) in canonical transcripts as specified by Ensembl.

We further performed rare variant association tests within the non-coding space (Supplementary Figure 7). As before, we performed a “sliding window” approach aggregating 3 kb (overlapping by 1.5 kb) windows and considering rare variants occurring within enhancer or promoter elements at DNase I hypersensitivity sites.

For non-coding tests, we next attempted to link rare non-coding variants with genes for association testing using regulatory annotations for HepG2 and adipose nuclei from ENCODE and NIH Roadmap. Given prior observations showing enrichment of functional promoter variants at *LIPG* with HDL-C extremes^52^, we similarly aggregated variants near TSSs. Prior studies have shown that approximately 80% of cis-eQTLs fall within 100 kb of TSS^53^. To increase the likelihood of mapping regulatory variants to the nearest gene, we were more restrictive and included variants overlapping promoter sequences ±5 kb and enhancer sequences ±20 kb of TSS at DNase I hypersensitivity sites.

We also linked chromatin state defined enhancers with genes using data from the Roadmap Epigenomics project^54^ and the method presented previously^55^ with a few small modifications^56^. The method predicts links using chromatin state information, position of the enhancer relative to the TSS, and the correlation of multiple chromatin marks with gene expression across cell types. Here we used the correlation with gene expression of the signal of five chromatin marks: H3K27ac, H3K9ac, H3K4me1, H3K4me2, and DNaseI hypersensitivity. The gene expression data were the RPKM expression data for protein-coding exons across 56 reference epigenomes from the Roadmap Epigenomics project (available in the file 57epigenomes.RPKM.pc from http://compbio.mit.edu/roadmap; Universal Human Reference was excluded). The chromatin mark signal was the −log_10_(*P*) tracks averaged to a 200-bp resolution. As input to our code, we used the version of those tracks first averaged at 25-bp resolution using the “Convert” command of ChromImpute^57^. In computing correlation between a specific chromatin mark signal and gene expression, we used the Pearson correlation and omitted from the calculation samples lacking both chromatin mark signal and gene expression data. We made predictions separately for each of the 127 reference epigenomes and locations assigned to chromatin states, 6_EnhG, 7_Enh, and 12_EnhBiv, of the 15-state core 5-marks ChromHMM model^54,58^. We restricted our predictions to chromatin state assignments on chr1-22 and chrX. We considered linking 200-bp bins within 1 Mb of a TSS of each gene as annotated in the file Ensembl_v65.Gencode_v10.ENSG.gene_info available from http://compbio.mit.edu/roadmap (ref. ^54^). If a gene had multiple TSS, then we only used the outermost TSS.

The method for linking is based on determining for each combination of cell type, chromatin state, and position relative to the TSS the estimated probability the set of correlations we observed would come from the actual data compared to randomized data. To this end, we created a training set of actual observed correlations (positive examples) and correlations computed after randomizing which gene expression values were assigned to which genes (negative examples) separately for each combination of cell type, chromatin state, and position relative to the TSS. Each entry in the training set has five features corresponding to correlations for each of the considered chromatin marks. There is a positive and a corresponding negative entry for each instance of the specified chromatin state in the specified cell type at the specified position relative to the TSS or within 5 kb of it (for smoothing purposes). We trained a logistic regression classifier to discriminate actual correlations with randomized correlations. We used the logistic regression library implemented in the Weka package version 3.7.3 with the regularization parameter set to 1^59^. For considering linking a specific instance of a chromatin state assignment in a specific cell type and position relative to the TSS of a gene, we applied the corresponding classifier. Let *p* denote the probability the classifier gives of being in the positive class of the actual observed correlations. We retained those links for which *p*/(1−*p*) was ≥2.5. The method we used here is implemented in the code LinkingRM.java. For the analyses presented here, we used those links for the primary enhancer state, 7_Enh.

To connect non-coding variants with putative target genes, we predicted functional gene-enhancer pairs using a chromatin state-based model we previously developed^15^. This model assumes that the impact of an enhancer on gene expression is determined by the product of its intrinsic “Activity” (for which we use quantitative DNase-Seq and H3K27ac ChIP-Seq levels as a proxy) and the “Contact Frequency” at which the enhancer physically encounters its target promoter in the nucleus (for which we use Hi-C data as a proxy). We previously found such an Activity by Contact (ABC) model accurately identifies enhancers whose perturbation leads to changes in gene expression in the human MYC locus^15^, and we have since found that the same model can identify enhancers across other gene loci and cell types (Fulco, C., Lander, E., and Engreitz, J., in preparation). We extended our previously published model to predict enhancer-gene connections in the liver, using DNase-Seq and H3K27ac ChIP-Seq data from a hepatocarcinoma cell line (HepG2) previously generated by the ENCODE project^60^. To define putative regulatory elements, we expanded DNase-Seq peak calls from ENCODE by 500 bp on either side and merged overlapping peaks^15^. For each element, we calculated Activity as a function of the normalized read count of H3K27ac and DNase-Seq. Because high-resolution Hi-C data is not available for HepG2 cells, we estimated the Contact probability between putative regulatory elements and genes using the average profile across deeply sequenced Hi-C libraries from seven different cell types^61^ as previously described^15^. For each putative enhancer-gene pair, we calculated an “ABC score” equal to the Activity × Contact of the putative enhancer normalized by the sum of Activity × Contact across all other putative elements within 5 Mb of the target gene. We tuned free parameters in this model (such as the relative weight of DNase-Seq and H3K27ac data and a pseudocount to add to Hi-C data) and chose a threshold cutoff using a set of experimentally measured enhancer–promoter connections in two cell types (Fulco, C., Lander, E., and Engreitz, J., in preparation). This analysis defined, for each expressed gene, a set of elements predicted to regulate that gene in HepG2 cells. These sets of elements were used for gene-level variant burden tests.

We tested the association of the aggregate MAF <1% variants within each of the aforementioned groupings with each lipid trait as continuous traits using the mixed-model SKAT implementation in EPACTS to account for bidirectional effects^11^. We first created group files (“make-group”) using annotations from the aforementioned strategies, created VCF-specific kinship matrices (“make-kin”) using default parameters, and performed association analyses (“group --test mmskat –max-maf 0.01”) (https://genome.sph.umich.edu/wiki/EPACTS). Analyses were adjusted for age, age^2^, sex, cohort, self-reported ethnicity (for MESA), and empiric kinship within each of the VCFs. *P* values for each grouping were meta-analyzed across the three callsets using Fisher’s method. Statistical significance for each gene-based test was 0.05/20,000 tests = 2.5 × 10^−6^.

### Lipid extremes analysis

We first defined LDL-C extremes as the top and bottom ancestry-specific 5th percentiles from the data (LDL-C >183 mg/dl or >198.6 mg/dl for EA and AA, respectively; LDL-C <72.9 mg/dl or <71 mg/dl for EA and AA, respectively).

We next cataloged mutations in Mendelian genes previously linked to extreme LDL-C (Supplementary Table 13). We included variants that were previously linked to Mendelian dyslipidemia in ClinVar (“pathogenic” or “likely pathogenic” with no “benign”) or loss-of-function, and had an allele frequency <1% (autosomal dominant) or <10% (autosomal recessive). Genotypes were only considered based on expected inheritance pattern (autosomal dominant or autosomal recessive).

We evaluated three distinct approaches to generate weighted polygenic scores using prior genome-wide association analysis summary statistics^7^: (1) only lead variants at genome-wide significant loci, (2) varying *P* and LD *r*^2^ thresholds (defined by 1000G CEU) using PLINK^62^, and (3) all variants but adjusting weights according to *P* and *r*^2^ (by 1000G CEU) with LDpred varying rho^16^. To minimize errors from strand flips, A/T and C/G SNPs were excluded. The scores were calculated as additive sums of risk allele counts for included SNPs multiplied by weights (discovery effect estimates for (1) and (2), or adjusted by LDpred for (3)).

LDPred^16^ is a Bayesian approach, calculates a posterior mean effect size for each variant based on a prior (association with LDL-C in a previously published study) and subsequent shrinkage based on the extent to which this variant is correlated with similarly associated variants in a reference population. The underlying Gaussian distribution additionally considers the fraction of causal (e.g., non-zero effect sizes) markers. Because this fraction is unknown for any given disease, LDpred uses a range of plausible values to construct different polygenic scores.

Polygenic scores were generated within the HUNT cohort, the training set^18^. Lipid values were extracted from the electronic health record; absence of lipid-lowering therapy was prioritized. For each trait, the model with the best fit, as measured by *R*^2^, was chosen to apply to the testing set, TOPMed samples.

In a multivariable model, we associated likelihood of membership within the extreme tail of a trait with monogenic mutation carrier status, high (top 5th percentile) or low (bottom 5th percentile) polygenic score, age, age^2^, and sex, separately in European American (EA from FHS and MESA-EA) and African American (AA from JHS and MESA-AA) samples. We also ran linear regression models with continuous LDL-C and the independent variables listed above.

### Data availability

Individual whole-genome sequence data for TOPMed whole genomes (FHS, JHS, OOA, and MESA) are available through restricted access via the TOPMed dbGaP Exchange Area. The accession numbers are: FHS phs000974.v1.p1, JHS phs000964.v1.p1, OOA phs000956.v1.p1, and MESA phs001416.v1.p1. Individual-level harmonized lipids used for analysis are also available through restricted access via the TOPMed dbGaP Exchange Area. Summary-level genotype data are available through the BRAVO browser (https://bravo.sph.umich.edu/). The Finnish WGS and array genotype data can be accessed through the THL Biobank (https://thl.fi/fi/web/thl-biobank). The WGS data at Estonian Genome Center, University of Tartu, can be accessed via the Estonian Biobank (www.biobank.ee).

## Electronic supplementary material

Supplementary Information

Peer Review File
