# Supplementary material for: Deep-coverage whole genome sequences and blood lipids among 16,324 individuals
Source: Nat Commun. 2018 Aug 23;9:3391. doi: 10.1038/s41467-018-05747-8 (PMC6107638; doi:10.1038/s41467-018-05747-8)
Supplement: Supplementary file 1 — Supplementary Information [file 41467_2018_5747_MOESM1_ESM.pdf]

Supplementary Information

Deep-coverage whole genome sequences and blood lipids among 16,324 individuals

Natarajan et al

Supplementary Note 1

Study participants

Framingham Heart Study (FHS)
The FHS is a three generational prospective cohort that has been described in detail previously.[1] Individuals were initially recruited in 1948 in Framingham, USA to evaluate cardiovascular disease risk factors. The second generation cohort (5,124 offspring of the original cohort) was recruited between 1971 and 1975. The third generation cohort (4,095 grandchildren of the original cohort) was collected between 2002 and 2005. Fasting lipid levels were measured at exam 1 of the Offspring (1971-1975) and third generation (2002-2005) cohorts, using standard LRC protocols.

Jackson Heart Study (JHS)
The JHS is a large, population-based observational study evaluating the etiology of cardiovascular, renal, and respiratory diseases among African Americans residing in the three counties (Hinds, Madison, and Rankin) that make up the Jackson, Mississippi metropolitan area.[2] Data and biologic materials have been collected from 5,301 participants, including a nested family cohort of 1,498 members of 264 families. The age at enrollment for the unrelated cohort was 35-84 years; the family cohort included related individuals >21 years old. Participants provided extensive medical and social history, had an array of physical and biochemical measurements and diagnostic procedures, and provided genomic DNA during a baseline examination (2000-2004) and two follow-up examinations (2005-2008 and 2009-2012). The study population is characterized by a high prevalence of diabetes, hypertension, obesity, and related disorders. Annual follow-up interviews and cohort surveillance are ongoing.

Old Order Amish (OOA)
The Old Order Amish individuals included in this study were participants of several ongoing studies of cardiovascular health carried out at the University of Maryland among relatively healthy volunteers from the Old Order Amish community of Lancaster County, PA and their family members.[3,4]

Multi-Ethnic Study of Atherosclerosis (MESA)
The Multi-Ethnic Study of Atherosclerosis[5] is a National Heart, Lung and Blood Institute-sponsored, population-based investigation of subclinical cardiovascular disease and its progression. A total of 6,814 individuals, aged 45 to 84 years, were recruited from six US communities (Baltimore City and County, MD; Chicago, IL; Forsyth County, NC; Los Angeles County, CA; New York, NY; and St. Paul, MN) between July 2000 and August 2002. Participants were excluded if they had physician-diagnosed cardiovascular disease prior to enrollment, including angina, myocardial infarction, heart failure, stroke or TIA, resuscitated cardiac arrest or a cardiovascular intervention (e.g., CABG, angioplasty, valve replacement, or pacemaker/defibrillator placement). Pre-specified recruitment plans identified four racial/ethnic groups (White European-American, African-American, Hispanic-American, and Chinese-American) for enrollment, with targeted oversampling of minority groups to enhance statistical power.

FINRISK (National FINRISK Study)

FINRISK was a population-based cross-sectional survey designed to study the prevalence of cardiovascular risk factors in Finland.[6] Surveys are conducted every 5 years, and the 1997 survey included 8,389 Finnish men and women aged 25-74. Participants underwent a physical examination and completed a questionnaire regarding cardiovascular risk factors. Of these FINRISK97 participants, 7,026 had DNA samples available for this analysis and data available for at least one lipoprotein or lipid phenotype.

EGCUT (Estonian Genome Center of University of Tartu)

The Estonian cohort is from the population-based biobank of the Estonian Genome Project of University of Tartu (EGCUT).[7] The project is conducted according to the Estonian Gene Research Act, and all participants have signed the broad informed consent. The current cohort size is > 51,515, 18 years of age and older, which reflects closely the age distribution in the adult Estonian population. Subjects are recruited by the general practitioners (GP) and physicians in the hospitals were randomly selected from individuals visiting GP offices or hospitals. Each participant filled out a computer-assisted personal interview during 1-2 hours at a doctor's office, including personal data (place of birth, place(s) of living, nationality. etc.), genealogical data (three generation family history), educational and occupational history, and lifestyle data (physical activity, dietary habits, smoking, alcohol consumption, women's health, quality of life).

Supplementary Note 2

Additional acknowledgements

The Framingham Heart Study has been supported by contracts N01-HC-25195 and HHSN268201500001I and grant R01 HL092577. The Framingham Heart Study thanks the study participants and the multitude of investigators who over its 70-year history continue to contribute so much to further our knowledge of heart, lung, blood and sleep disorders and associated traits. The Jackson Heart Study (JHS) is supported and conducted in collaboration with Jackson State University (HHSN268201300049C and HHSN268201300050C), Tougaloo College (HHSN268201300048C), and the University of Mississippi Medical Center (HHSN268201300046C and HHSN268201300047C) contracts from the National Heart, Lung, and Blood Institute (NHLBI) and the National Institute for Minority Health and Health Disparities (NIMHD). The authors also wish to thank the staffs and participants of the JHS. J.G.W. is supported by U54GM115428 from the National Institute of General Medical Sciences.

MESA and the MESA SHARe project are conducted and supported by the National Heart, Lung, and Blood Institute (NHLBI) in collaboration with MESA investigators. Support for MESA is provided by contracts HHSN268201500003I, N01-HC-95159, N01-HC-95160, N01-HC-95161, N01-HC-95162, N01-HC-95163, N01-HC-95164, N01-HC-95165, N01-HC-95166, N01-HC-95167, N01-HC-95168, N01-HC-95169, UL1-TR-000040, UL1-TR-001079, UL1-TR-001420. The provision of genotyping data was supported in part by the National Center for Advancing Translational Sciences, CTSI grant UL1TR001881, and the National Institute of Diabetes and Digestive and Kidney Disease Diabetes Research Center (DRC) grant DK063491 to the Southern California Diabetes Endocrinology Research Center.

Supplementary Table 1. Samples filtered by quality control metrics

	OOA	FHS	JHS	MESA	FIN	EST
Contamination*	6	20	1	66	8	12
Chimeras > 5%	0	0	0	0	4	0
GC dropout > 4	0	0	0	0	1	0
Raw coverage†	0	7	0	23	0	4
Indeterminate genotypic sex‡	0	0	0	1	2	0
Duplicates / monozygotic twins§	4	15	2	1	0	0
Expected population outliers by PCA	0	5	14	0	0	0
Variant metric count outliers	0	6	2	0	0	0
Array-sequencing genotype concordance < 0.95	0	1	0	0	0	10
Mendelian violations	2	0	0	0	0	0
TOTAL FILTERED	12	50	19	91	15	26

* Contamination threshold for all samples was > 3.0% but was >5.0% for the combined Finland and Estonia callset.
† Raw coverage threshold for all samples was <30X but was <19X for the combined Finland and Estonia callset.
‡ Chromosome X F inbreeding coefficient 0.5-0.8 was used to denote indeterminate genotypic sex.
§ Duplicates / monozygote twins were identified by identity-by-descent (PI HAT) > 0.95.
Filtering for phase 1 TOPMed samples (OOA, FHS, and JHS) may be slightly under-reported as sequencing centers may have filtered samples prior to transfer to the TOPMed Informatics Research Core.
EST = Estonia, FHS = Framingham Heart Study, FIN = Finland, JHS = Jackson Heart Study, MESA = Multi-Ethnic Study of Atherosclerosis, OOA = Old Order Amish, TOPMed = Trans-Omics for Precision Medicine

Supplementary Table 2. Baseline characteristics of study participants

	OOA	FHS	JHS	MESA	FIN	EST
N	1,083	4,064	3,247	4,510	1,165	2,255
Coverage – X	38.3 (6.6)	37.7 (6.1)	37.6 (4.8)	35.7 (4.5)	30.1 (3.4)	29.7 (5.7)
Age – y	50 (17)	40 (11)	56 (13)	61 (10)	49 (13)	47 (18)
Women	535 (49%)	2,198 (54%)	2,043 (63%)	2,193 (49%)	596 (51%)	1,104 (49%)
European ancestry	1,083 (100%)	4,064 (100%)	0 (0%)	1,846 (41%)	1,165 (100%)	2,255 (100%)
Cholesterol – mg/dl						
Total	212 (47)	198 (39)	199 (41)	195 (35)	216 (41)	215 (47)
LDL	140 (43)	121 (35)	126 (37)	118 (31)	133 (35)	107 (35)
HDL	56 (16)	54 (16)	52 (15)	51 (15)	55 (15)	59 (16)
Triglycerides – mg/dl*	63 [46-96]	89 [61-143]	90 [64-128]	112 [78-162]	111 [80-166]	119 [86-166]
Blood pressure – mmHg						
Systolic	121 (16)	120 (16)	127 (18)	125 (21)	135 (19)	129 (19)
Diastolic	74 (10)	77 (10)	79 (11)	72 (10)	81 (11)	80 (12)
Diabetes mellitus	26 (2.4%)	67 (2%)	701 (22%)	382 (9%)	193 (16%)	89 (4%)
Body-mass index	27 (5)	26 (5)	32 (7)	28 (5)	27 (5)	27 (5)
Current smoker	106 (10%)	1,143 (29%)	399 (12%)	559 (12%)	382 (32%)	254 (5%)
Current drug therapy						
Statins	35 (3%)	167 (4%)	405 (13%)	724 (16%)	56 (5%)	113 (5%)
Antihypertensive	54 (5%)	248 (6%)	1,654 (51%)	1,424 (32%)	161 (14%)	601 (27%)

Counts – N (%)
Continuous – Mean (Standard Deviation)
* Triglycerides are summarized as median [IQR]
EST = Estonia, FHS = Framingham Heart Study, FIN = Finland, HDL = high-density lipoprotein, JHS = Jackson Heart Study, LDL = low-density lipoprotein, MESA = Multi-Ethnic Study of Atherosclerosis, OOA = Old Order Amish

Supplementary Table 3. Distributions of variant metrics per sample.

Call Set	Cohort	Transitions / Transversions	Heterozygous / Homozygous genotypes	Total Variants per Sample	Singletons per Sample
TOPMed Phase 1	OOA	2.153<br>[2.152-2.153]	1.46<br>[1.43-1.49]	3,324,000<br>[3309000-3337000]	90<br>[62-194]
	FHS	2.152<br>[2.151-2.153]	1.56<br>[1.55-1.56]	3,363,000<br>[3352000-3376000]	2,518<br>[893-4,556]
	JHS	2.148<br>[2.147-2.148]	2.07<br>[2.05-2.10]	4,086,000<br>[4049000-4111000]	8,205<br>[4,396-10,540]
MESA	MESA				
	EUR	2.127<br>[2.125-2.128]	1.64<br>[1.63-1.64]	2,983,000<br>[2974000-2994000]	10,880<br>[10,100-11,750]
	AFR	2.123<br>[2.121-2.124]	2.12<br>[2.10-2.14]	3,519,000<br>[3461000-3557000]	16,470<br>[15,510-17,450]
	ASN	2.119<br>[2.117-2.120]	1.38<br>[1.37-1.39]	2,881,000<br>[2876000-2887000]	20,360<br>[18,590-21,990]
	HISP	2.123<br>[2.121-2.124]	1.66<br>[1.60-1.69]	3,011,000<br>[2986000-3090000]	14,430<br>[11,240-15,570]
Finland & Estonia	FIN	2.127<br>[2.126-2.128]	1.51<br>[1.49-1.52]	3,596,000<br>[3588000-3606000]	3,130<br>[2,298-4,276]
	EST	2.127<br>[2.125-2.128]	1.51<br>[1.50-1.53]	3,597,000<br>[3585000-3607000]	4,389<br>[3,793-5,054]
ALL		2.146<br>[2.125-2.151]	1.58<br>[1.52-2.03]	3,391,000<br>[3329000-3607000]	4,878<br>[2,512-10,830]

AFR = African ancestry, ASN = Asian ancestry, EST = Estonia, EUR = European ancestry, FHS = Framingham Heart Study, FIN = Finland, JHS = Jackson Heart Study, HISP = Hispanic ancestry, MESA = Multi-Ethnic Study of Atherosclerosis, OOA = Old Order Amish

Supplementary Table 4. Genomic inflation metrics for single variant association analyses.

λ_{GC}	MAF 0.1-0.5%	MAF 0.5-1%	MAF 1-5%	MAF 5-50%
Total cholesterol	1.054	1.073	1.076	1.031
HDL-C	0.981	0.983	0.987	1.023
LDL-C	1.055	1.076	1.083	1.023
Triglycerides	0.959	0.943	0.946	1.013

HDL-C = high-density lipoprotein cholesterol, LDL-C = low-density lipoprotein cholesterol, MAF = minor allele frequency, λ_{GC} = genomic control lambda metric

Supplementary Table 5. Allele frequency distribution of variants associated ($P < 5x10^{-8}$) with plasma lipids.

N	MAF 0.1-0.5%	MAF 0.5-1%	MAF 1-5%	MAF 5-50%	ALL
Total cholesterol	16	13	109	454	592
HDL-C	2	1	17	427	447
LDL-C	28	25	106	538	697
Triglycerides	4	1	13	504	522

HDL-C = high-density lipoprotein cholesterol, LDL-C = low-density lipoprotein cholesterol, MAF = minor allele frequency

Supplementary Table 6. Lead individual genome sequence variants associated with blood lipids.

Locus	Genomic coordinates*	rsID	Gene, consequence†	Alternate allele frequency, Callsets with MAF>0.1%‡	Alternate allele effect estimate	Standard error	*P*	Known locus§	Prior lead‖	Lead previously tagged¶
Total cholesterol										
19q13.32	19:45412079:C:T	rs7412	*APOE* p.Arg176Cys	8.0 % T, M, F	-15.4 mg/dL	0.8 mg/dL	8.1x10^{-94}	Y	Y	Y
19p13.2	19:11190652:G:A	rs142130958	(*LDLR-DOCK6*)	11.6 % T, M, F	-10.7 mg/dL	0.7 mg/dL	6.2x10^{-50}	Y	N	Y
1p32.3	1:55529215:C:A	rs28362286	*PCSK9* p.Cys679Ter	0.4 % T, M	-39.9 mg/dL	4.1 mg/dL	4.5x10^{-27}	Y	N	N
1p13.3	1:109817590:G:T	rs12740374	(*SORT1*)	23.4 % T, M, F	-5.4 mg/dL	0.5 mg/dL	9.6x10^{-25}	Y	Y	Y
2p24.1	2:21267461:G:A	rs934197	(*APOB*)	24.7 % T, M, F	+4.3 mg/dL	0.5 mg/dL	2.6x10^{-16}	Y	N	Y
19p13.11	19:19329924:C:T	rs2228603	*NCAN* p.Pro92Thr	5.5 % T, M, F	-6.0 mg/dL	1.0 mg/dL	1.7x10^{-10}	Y	N	Y
16q22.2	16:72188889:G:A	rs60201663	(*PMFBP1*)	4.4 % T, M	+7.5 mg/dL	1.3 mg/dL	1.0x10^{-9}	Y	N	N
2p21	2:44074431:C:T	rs4245791	(*ABCG8*)	75.6 % T, M, F	-3.2 mg/dL	0.5 mg/dL	2.6x10^{-9}	Y	N	Y
2p23.3	2:27730940:T:C	rs1260326	*GCKR* p.Leu446Pro	66.5 % T, M, F	-2.9 mg/dL	0.5 mg/dL	6.4x10^{-9}	Y	Y	Y
17q11.1	17:25686461:G:A	rs74858876	(*WSB1*)	0.9 % T, M, F	-13.5 mg/dL	2.3 mg/dL	1.2x10^{-8}	N	N	N
17p12	17:15328696:A:C	NA	(*TVP23C-CDRT4*)	0.7% F	+42.7 mg/dL	6.8 mg/dL	2.5x10^{-8}	N	N	N
5q33.2	5:152714715:T:A	rs186120725	(*GRIA1*)	1.1 % T, M, F	+13.1 mg/dL	2.3 mg/dL	2.8x10^{-8}	N	N	N
15q21.3	15:58723675:C:T	rs1800588	(*LIPC*)	31.4 % T, M, F	+2.8 mg/dL	0.5 mg/dL	3.9x10^{-8}	Y	Y	Y
LDL-C										
19q13.32	19:45412079:C:T	rs7412	*APOE* p.Arg176Cys	8.0 % T, M, F	-19.6 mg/dL	0.8 mg/dL	3.6x10^{-186}	Y	Y	Y
19p13.2	19:11190652:G:A	rs142130958	(*LDLR-DOCK6*)	11.6 % T, M, F	-10.5 mg/dL	0.7 mg/dL	1.3x10^{-57}	Y	N	Y
1p32.3	1:55529215:C:A	rs28362286	*PCSK9* p.Cys679Ter	0.4 % T, M	-43.0 mg/dL	3.8 mg/dL	3.7x10^{-36}	Y	N	N
1p13.3	1:109817590:G:T	rs12740374	(*SORT1*)	23.4 % T, M, F	-5.9 mg/dL	0.5 mg/dL	1.6x10^{-33}	Y	Y	Y
2p24.1	2:21267461:G:A	rs934197	(*APOB*)	25.0 % T, M, F	+4.3 mg/dL	0.5 mg/dL	1.4x10^{-18}	Y	Y	Y
2p21	2:44074431:C:T	rs4245791	(*ABCG8*)	75.6 % T, M, F	-3.1 mg/dL	0.5 mg/dL	2.4x10^{-10}	Y	N	Y
10q25.2	10:113028253:A:G	rs779261368	(*GPAM*)	0.3 % F	+57.6 mg/dL	8.5 mg/dL	4.9x10^{-9}	Y	N	N
7q31.33	7:124373018:T:A	rs185350393	(*GPR37*)	0.4 % T, M	-20.2 mg/dL	3.8 mg/dL	1.8x10^{-8}	N	N	N
19q12	19:31902141:G:A	rs138573424	(*TSHZ3*)	0.6 % T, M	+17.0 mg/dL	3.1 mg/dL	2.1x10^{-8}	N	N	N
17q11.1	17:25686461:G:A	rs74858876	(*WSB1*)	0.9 % T, M, F	-12.5 mg/dL	2.2 mg/dL	2.9x10^{-8}	N	N	N
HDL-C										
16q13	16:56990716:C:A	rs247617	(*CETP*)	29.5 % T, M, F	+3.3 mg/dL	0.2 mg/dL	6.2x10^{-85}	Y	Y	Y
8p21.3	8:19819439:A:G	rs326	(*LPL*)	37.8 % T, M, F	+1.7 mg/dL	0.2 mg/dL	4.0x10^{-24}	Y	N	N
15q21.3	15:58674308:G:A	rs2043082	(*LIPC*)	31.8 % T, M, F	+1.5 mg/dL	0.2 mg/dL	1.7x10^{-19}	Y	N	Y
11q23.3	11:116623213:TA:T	rs66505542	(*APOA1-APOC3-APOA5*)	77.5 % T, M, F	+1.3 mg/dL	0.2 mg/dL	1.2x10^{-12}	Y	N	Y
19p13.2	19:8429323:G:A	rs116843064	*ANGPTL4* p.Glu40Lys	1.6 % T, M, F	+4.0 mg/dL	0.6 mg/dL	6.1x10^{-11}	Y	Y	Y
19p13.2	19:11350488:C:T	rs2278426	*ANGPTL8* p.Arg59Trp (*LDLR-DOCK6*)	10.0 %	-1.7 mg/dL	0.3 mg/dL	1.4x10^{-10}	Y	N	N
18q21.1	18:47167214:T:C	rs4939883	(*LIPG*)	74.8 % T, M, F	+1.1 mg/dL	0.2 mg/dL	1.9x10^{-10}	Y	Y	Y

9q31.1	9:107664301:C:T	rs1883025	(*ABCA1*)	26.7 % T, M, F	-1.1 mg/dL	0.2 mg/dL	$2.2x10^{-9}$	Y	Y	Y
12q24.31	12:125338529:C:T	rs10773112	(*SCARB1*)	63.1 % T, M, F	+0.9 mg/dL	0.2 mg/dL	$6.2x10^{-9}$	Y	N	N
1q42.3	1:229336103:A:C	NA	(*GALNT2*)	0.2% F	+28.8 mg/dL	4.3 mg/dL	$8.9x10^{-9}$	Y	N	N
9p24.1	9:5436973:TA:T	rs3215707	(*PLGRKT*)	2.0 % T, M, F	+3.3 mg/dL	0.6 mg/dL	$1.3x10^{-8}$	Y	N	N
19q13.32	19:45392254:C:T	rs6857	(*APOE*)	13.2 % T, M, F	-1.3 mg/dL	0.2 mg/dL	$1.6x10^{-8}$	Y	N	N
19q13.41	19:52296537:A:C	rs17834799	(*HAS1*)	18.4 % T, M, F	+1.1 mg/dL	0.2 mg/dL	$2.6x10^{-8}$	Y	N	N
ln(Triglycerides)										
11q23.3	11:116623213:TA:T	rs66505542	(*APOA1-APOC3-APOA5*)	77.5 % T, M, F	-0.093	0.007	$4.7x10^{-35}$	Y	N	Y
8p21.3	8:19819439:A:G	rs326	(*LPL*)	37.8 % T, M, F	-0.074	0.007	$3.3x10^{-30}$	Y	N	N
2p23.3	2:27730940:T:C	rs1260326	*GCKR* p.Leu446Pro	66.5 % T, M, F	-0.060	0.007	$9.9x10^{-21}$	Y	Y	Y
19p13.2	19:8429323:G:A	rs116843064	*ANGPTL4* p.Glu40Lys	1.6 % T, M, F	-0.168	0.023	$3.9x10^{-14}$	Y	Y	Y
19q13.32	19:45422587:A:G	rs12721054	(*APOE-APOC1*)	4.0 % T, M	-0.132	0.018	$9.0x10^{-14}$	Y	Y	Y
8q24.13	8:126488930:A:G	rs2980871	(*TRIB1*)	33.6 % T, M, F	-0.046	0.007	$2.3x10^{-11}$	Y	N	Y
7q11.23	7:73016862:T:C	rs13240994	(*MLXIPL*)	15.5 % T, M, F	-0.053	0.009	$2.8x10^{-10}$	Y	N	Y
17q21.31	17:41926126:C:T	rs72836561	*CD300LG* p.Arg82Cys	1.7 % T, M, F	+0.132	0.022	$2.5x10^{-9}$	Y	N	Y
19p13.11	19:19578743:A:G	rs73002956	(*CILP2*)	84.4 % M, F	-0.065	0.011	$1.2x10^{-8}$	Y	Y	Y

* hg19 reference genome. Chromosome:Position:Reference Allele:Alternate Allele format

† Nearest mapped gene listed in parenthesis when lead variant occurs outside of exonic regions. Consequence is listed for variants occurring within exonic regions.

‡ Alternate allele frequency is calculated from callsets where minor allele frequency exceeded 0.1%. Callsets that achieved this are indicated as T (TOPMed Phase 1), M (MESA), or F (Finland and Estonia).

§ Locus is previously significantly ($P < 5 \times 10^{-8}$) associated with, ‖ is prior lead variant in significantly associated locus from, or ¶ in linkage disequilibrium ($r^2 > 0.8$) with prior lead variant at locus in prior lipids genome-wide association studies.[8-11]

Using a conventional alpha threshold of $P<5x10^{-8}$, 13, 10, 13, and 9 loci associated with total cholesterol, LDL-C, HDL-C, and triglycerides, respectively, including 5 at putative novel lipid loci. Using a more stringent alpha of $P<5x10^{-9}$,[12] 8, 7, 8, and 8 loci associated with total cholesterol, LDL-C, HDL-C, and triglycerides, respectively, which were all at known lipid loci. However, of these lead variants attaining $P<5x10^{-9}$, 2, 2, 2, and 1 variants were not previously tagged by prior lead variants.

MAF = minor allele frequency; TOPMed = Trans-Omics for Precision Medicine

Supplementary Table 7. Association of rs3215707 with HDL cholesterol by call set and meta-analyzed results.

Callset	MAF	Effect estimate (mg/dL)	Standard error (mg/dL)	*P*
TOPMed Phase I	1.7 %	+2.7	0.9	2.0×10^{-3}
MESA	1.7%	+2.8	1.1	6.4×10^{-3}
Finland & Estonia	3.2%	+4.7	1.1	4.4×10^{-5}
All*	2.2 %	+3.3	0.6	1.3×10^{-8}

* Meta-analyzed results

Supplementary Table 8. Conditional single variant analyses among 8,394 TOPMed participants.

Locus	Mapped gene	Known locus	Independent associations with $P < 5x10^{-8}$	Independent associations with $P < 1x10^{-4}$
LDL-C				
1p32.3	*PCSK9*	Y	3<br>rs28362286<br>rs74073082<br>rs11591147	7
1p13.3	*SORT1-CELSR2*	Y	1<br>rs12740374	2
1q24.2	NA	N	1<br>rs116593889	1
2p24.1	*APOB*	Y	2<br>rs5742904<br>rs1041968	5
8q24.12	NA	N	1<br>rs77049078	1
19p13.2	*LDLR-DOCK6*	Y	1<br>rs138294113	7
19q12	NA	N	1<br>rs151327765	2
19q13.32	*APOE-APOC1*	Y	3<br>rs7412<br>rs429358<br>rs114083252	6
HDL-C				
8p21.3	*LPL*	Y	1<br>rs326	3
15q21.3	*LIPC*	Y	2<br>rs2070895<br>rs2043082	2
16q13	*CETP*	Y	3<br>rs247616<br>rs2033254<br>rs5883	7
Triglycerides				
1q43	*RYR2*	N	1<br>rs541184829	4
2p23.3	*GCKR*	Y	1<br>rs1260326	1
8p21.3	*LPL*	Y	1<br>rs326	3
11q23.3	*APOC3*	Y	1<br>rs66505542	5
19q13.32	*APOE-APOC1*	Y	1<br>rs12721054	3

All associations were adjusted for age, age^2, sex, and empiric kinship matrix. LDL-C associations were further adjusted for *APOB* p.Arg3527Gln and HDL-C and triglyceride associations were further adjusted for *APOC3* p.Arg19Ter. The rsIDs of independent variants attaining $P < 5x10^{-8}$ are displayed.

Supplementary Table 9. Mixed model SKAT association of disruptive protein-coding mutations and blood lipids.

Gene	cMAF	*P*
LDL-C		
LDLR	0.021	$3.4x10^{-9}$
APOB	0.026	$2.1x10^{-7}$
PCSK9	0.025	$3.2x10^{-42}$
LDLRAP1	0.0015	0.38
ABCG5	0.012	0.12
ABCG8	0.033	0.071
APOE	0.019	$7.2x10^{-10}$
HDL-C		
APOA1	0	NA
ABCA1	0.023	$1.3x10^{-3}$
LCAT	0.0026	$1.6x10^{-7}$
CETP	0.0013	$7.6x10^{-5}$
LIPC	0.014	0.16
LIPG	0.0024	0.29
SCARB1	0.0056	$3.6x10^{-4}$
Triglycerides		
LPL	0.0042	$7.9x10^{-5}$
APOC2	0	NA
APOA5	0	NA
APOC3	0.0089	$1.1x10^{-14}$
GPIHBP1	0	NA
LMF1	0.0033	0.29
ANGPTL3	0.0024	0.032
ANGPTL4	0.0029	0.026

Genes without disruptive mutations have cMAF 0 and P = NA.
cMAF = combined minor allele frequency

Supplementary Table 10. Characteristics of grouping strategies for rare variant association testing.

	Tissue type	Groups		TOPMed phase 1	MESA	Finland & Estonia
Coding (LOF + missense metaSVM)	NA	11,786	cMAF	0.24 [0.08-0.73] %	0.29 [0.11-0.82] %	0.15 [0.058-0.41] %
			n variants	6 [3-11]	6 [3-13]	2 [1-4]
Sliding window	NA	180,240	cMAF	2.5 [1.1-4.4] %	2.8 [1.3-4.9] %	0.8 [0.3-1.9] %
			n variants	22 [12-38]	24 [14-42]	6 [3-11]
Transcription start site proximity	HepG2	18,887	cMAF	7.3 [3.8-11.9] %	8.1 [4.4-13.2] %	3.0 [1.4-5.4] %
			n variants	70 [38-119]	79 [43-134]	21 [11-36]
	Adipose nuclei	19,137	cMAF	7.3 [3.8-12.0] %	8.1 [4.3-13.2] %	3.0 [1.4-5.5] %
			n variants	70 [37-119]	78 [42-134]	21 [11-36]
Gene expression	HepG2	12,941	cMAF	6.2 [2.4-14.0] %	6.9 [2.6-15.3] %	2.5 [0.8-6.2] %
			n variants	55 [21-137]	62 [24-155]	17 [6-42]
	Adipose nuclei	12,869	cMAF	4.8 [1.9-11.3] %	5.4 [2.2-12.5] %	2.0 [0.6-5.1] %
			n variants	42 [17-107]	48 [19-120]	13 [5-32]
Hi-C	NA	9,958	cMAF	22.4 [16.3-29.5] %	25.0 [18.4-32.6] %	11.0 [7.3-15.2] %
			n variants	263 [182-367]	298 [208-417]	78 [54-110]

Summary statistics provided for groups that were included in meta-analysis. If at least one callset had >1 variant with MAF<1% meeting inclusion criteria within a group, the group was included in analysis.

Supplementary Table 11. mmSKAT association of rare coding and non-coding gene-linked variants and blood lipids.

	P value					
Gene	LoF + MetaSVM-D	TSS Proximity, HepG2	TSS Proximity, Adipose nuclei	Gene expression, HepG2	Gene expression, Adipose nuclei	HiC
LDL-C						
LDLR	3.4×10^{-9}	9.7×10^{-11}	3.6×10^{-10}	9.2×10^{-10}	0.22	2.2×10^{-9}
APOB	2.1×10^{-7}	7.1×10^{-3}	5.3×10^{-4}	0.09	4.6×10^{-5}	7.9×10^{-4}
PCSK9	3.2×10^{-42}	6.7×10^{-4}	1.1×10^{-3}	4.3×10^{-4}	7.7×10^{-4}	9.1×10^{-4}
LDLRAP1	0.38	NA	0.53	0.21	0.55	0.25
ABCG5	0.12	NA	NA	4.7×10^{-2}	0.13	0.20
ABCG8	0.071	NA	NA	2.7×10^{-2}	6.9×10^{-2}	0.20
APOE	7.2×10^{-10}	1.3×10^{-3}	5.3×10^{-4}	8.1×10^{-26}	9.2×10^{-7}	8.7×10^{-13}
HDL-C						
APOA1	NA	NA	0.80	0.35	6.2×10^{-2}	0.28
ABCA1	1.3×10^{-3}	3.0×10^{-2}	5.3×10^{-3}	0.11	7.1×10^{-2}	7.2×10^{-2}
LCAT	1.6×10^{-7}	0.17	5.0×10^{-2}	3.6×10^{-2}	0.14	0.18
CETP	7.6×10^{-5}	4.7×10^{-3}	6.0×10^{-2}	2.9×10^{-3}	5.1×10^{-5}	NA
LIPC	0.16	6.7×10^{-3}	2.8×10^{-3}	1.4×10^{-2}	2.6×10^{-2}	2.6×10^{-2}
LIPG	0.29	0.32	0.28	0.22	0.47	0.11
SCARB1	3.6×10^{-4}	1.2×10^{-3}	0.17	6.5×10^{-2}	0.30	0.20
Triglycerides						
LPL	7.9×10^{-5}	4.2×10^{-4}	3.6×10^{-3}	NA	1.8×10^{-2}	NA
APOC2	NA	0.31	0.84	0.16	8.9×10^{-3}	1.6×10^{-2}
APOA5	NA	0.62	0.48	5.6×10^{-3}	1.6×10^{-2}	0.20
APOC3	1.1×10^{-14}	7.8×10^{-2}	NA	0.12	3.0×10^{-2}	0.13
GPIHBP1	NA	NA	2.7×10^{-2}	NA	NA	NA
LMF1	0.29	4.1×10^{-2}	0.28	NA	NA	0.19
ANGPTL3	0.032	NA	NA	0.47	NA	0.48
ANGPTL4	0.026	2.8×10^{-3}	2.8×10^{-3}	0.35	0.41	0.33

Supplementary Table 12. Coding and non-coding rare variant association analyses for *LDLR*, *APOB*, *PCSK9*, and *APOE*.

Annotation	Number of variants included	SKAT-O *P*	Gene	Analysis type
DHS	420	2.20E-13	*LDLR*	Non-adjusted
CADD > 95th perc	85	0.016507761	*LDLR*	Non-adjusted
Damaging	70	0.000105029	*LDLR*	Non-adjusted
Synonymous	64	0.501905747	*LDLR*	Non-adjusted
Introns	2554	8.84E-10	*LDLR*	Non-adjusted
By gene-express	147	1.16E-06	*LDLR*	Non-adjusted
By distance	353	1.30E-06	*LDLR*	Non-adjusted
By Hi-C	323	6.09E-06	*LDLR*	Non-adjusted
DHS	244	0.000821988	*PCSK9*	Non-adjusted
CADD > 95th perc	116	9.45E-07	*PCSK9*	Non-adjusted
Damaging	27	1.29E-49	*PCSK9*	Non-adjusted
Synonymous	61	0.012308808	*PCSK9*	Non-adjusted
Introns	1235	1.23E-05	*PCSK9*	Non-adjusted
By gene-express	149	0.001039663	*PCSK9*	Non-adjusted
By distance	96	0.000336438	*PCSK9*	Non-adjusted
By Hi-C	195	0.000837411	*PCSK9*	Non-adjusted
DHS	180	0.248127596	*APOB*	Non-adjusted
CADD > 95th perc	84	0.505270678	*APOB*	Non-adjusted
Damaging	32	7.98E-81	*APOB*	Non-adjusted
Synonymous	264	0.379767073	*APOB*	Non-adjusted
Introns	1662	0.007298343	*APOB*	Non-adjusted
By gene-express	2046	1.12E-16	*APOB*	Non-adjusted
By distance	160	0.171355299	*APOB*	Non-adjusted
By Hi-C	443	4.77E-39	*APOB*	Non-adjusted
DHS	284	0.000442443	*APOE*	Non-adjusted
CADD > 95th perc	174	1.35E-06	*APOE*	Non-adjusted
Damaging	20	3.35E-12	*APOE*	Non-adjusted
Synonymous	20	0.230403594	*APOE*	Non-adjusted
Introns	115	3.63E-12	*APOE*	Non-adjusted
By gene-express	1682	4.14E-24	*APOE*	Non-adjusted
By distance	140	0.001408016	*APOE*	Non-adjusted
By Hi-C	725	2.39E-15	*APOE*	Non-adjusted
DHS	420	1.58E-13	*LDLR*	Adjusted by damaging
CADD > 95th perc	85	0.01808748	*LDLR*	Adjusted by damaging
By gene-express	147	1.17E-07	*LDLR*	Adjusted by damaging
By Hi-C	323	8.67E-06	*LDLR*	Adjusted by damaging
DHS	244	0.00018199	*PCSK9*	Adjusted by damaging
CADD > 95th perc	116	2.82E-06	*PCSK9*	Adjusted by damaging
By gene-express	149	0.000432226	*PCSK9*	Adjusted by damaging
By Hi-C	195	0.000913643	*PCSK9*	Adjusted by damaging
DHS	180	0.391618489	*APOB*	Adjusted by damaging
CADD > 95th perc	84	0.63418993	*APOB*	Adjusted by damaging
By gene-express	2046	0.014207386	*APOB*	Adjusted by damaging
By Hi-C	443	0.036077219	*APOB*	Adjusted by damaging
DHS	284	0.000364942	*APOE*	Adjusted by damaging
CADD > 95th perc	174	1.48E-06	*APOE*	Adjusted by damaging
By gene-express	1682	1.38E-11	*APOE*	Adjusted by damaging
By Hi-C	725	6.13E-09	*APOE*	Adjusted by damaging
DHS	420	8.42E-05	*LDLR*	ENGAGE significant hits
CADD > 95th perc	85	0.01184684	*LDLR*	ENGAGE significant hits
Damaging	70	1.60E-05	*LDLR*	ENGAGE significant hits

By gene-express	147	0.060336229	*LDLR*	ENGAGE significant hits
By Hi-C	323	0.002780276	*LDLR*	ENGAGE significant hits
DHS	244	0.153720085	*PCSK9*	ENGAGE significant hits
CADD > 95th perc	116	0.002863322	*PCSK9*	ENGAGE significant hits
Damaging	27	1.05E-40	*PCSK9*	ENGAGE significant hits
By gene-express	149	0.016102622	*PCSK9*	ENGAGE significant hits
By Hi-C	195	0.033758681	*PCSK9*	ENGAGE significant hits
DHS	180	0.797426031	*APOB*	ENGAGE significant hits
CADD > 95th perc	84	0.37793502	*APOB*	ENGAGE significant hits
Damaging	32	7.66E-57	*APOB*	ENGAGE significant hits
By gene-express	2046	6.46E-11	*APOB*	ENGAGE significant hits
By Hi-C	443	7.98E-25	*APOB*	ENGAGE significant hits
DHS	284	0.537612708	*APOE*	ENGAGE significant hits
CADD > 95th perc	174	0.761014142	*APOE*	ENGAGE significant hits
Damaging	20	1.52E-05	*APOE*	ENGAGE significant hits
By gene-express	1682	0.007776608	*APOE*	ENGAGE significant hits
By Hi-C	725	0.068487884	*APOE*	ENGAGE significant hits
DHS	420	0.029475065	*LDLR*	ENGAGE + WGS significant hits
CADD > 95th perc	85	0.395846423	*LDLR*	ENGAGE + WGS significant hits
Damaging	70	1.68E-05	*LDLR*	ENGAGE + WGS significant hits
By gene-express	147	0.777551136	*LDLR*	ENGAGE + WGS significant hits
By Hi-C	323	0.192301922	*LDLR*	ENGAGE + WGS significant hits
DHS	244	0.06686142	*PCSK9*	ENGAGE + WGS significant hits
CADD > 95th perc	116	0.002151819	*PCSK9*	ENGAGE + WGS significant hits
Damaging	27	1.29E-05	*PCSK9*	ENGAGE + WGS significant hits
By gene-express	149	0.009146326	*PCSK9*	ENGAGE + WGS significant hits
By Hi-C	195	0.042111119	*PCSK9*	ENGAGE + WGS significant hits
DHS	180	1	*APOB*	ENGAGE + WGS significant hits
CADD > 95th perc	84	0.846066671	*APOB*	ENGAGE + WGS significant hits
Damaging	32	0.448266106	*APOB*	ENGAGE + WGS significant hits
By gene-express	2046	0.85995284	*APOB*	ENGAGE + WGS significant hits
By Hi-C	443	0.795058406	*APOB*	ENGAGE + WGS significant hits
DHS	284	0.683875324	*APOE*	ENGAGE + WGS significant hits
CADD > 95th perc	174	1	*APOE*	ENGAGE + WGS significant hits
Damaging	20	0.617432871	*APOE*	ENGAGE + WGS significant hits
By gene-express	1682	0.87541804	*APOE*	ENGAGE + WGS significant hits
By Hi-C	725	0.834482658	*APOE*	ENGAGE + WGS significant hits

Supplementary Table 13. Genes and monogenic mutations considered as contributors to extreme lipid phenotypes.

Monogenic risk genotypes:
1. AD = 0/1 or 1/1; AR = 1/1
2. MAF < 1% (AD) or <10% (AR)
3. ClinVar[13] LP/P for expected phenotype and not B
   or "high confidence" loss-of-function by LOFTEE[14])

Phenotype	Gene	Inheritance	Exceptions
High LDL-C	*LDLR*	AD	
	APOB	AD	*only* ClinVar "familial_hypercholesterolemia"
	PCSK9	AD	*only* ClinVar "familial_hypercholesterolemia"
	LDLRAP1	AR	
	ABCG5	AR	
	ABCG8	AR	
Low LDL-C	*APOB*	AD	*except* ClinVar "familial_hypercholesterolemia"
	PCSK9	AD	*except* ClinVar "familial_hypercholesterolemia"

AD = autosomal dominant; AR = autosomal recessive; HDL = high-density lipoprotein; LDL-C = low-density lipoprotein cholesterol; LP = likely pathogenic; MAF = minor allele frequency; P = pathogenic

Supplementary Table 14. Baseline characteristics of unrelated individuals within the HUNT cohort with lipids.

N	25,534
Female	13,587 (53%)
Age (y)	48 (16)
Diabetes mellitus, type 2	1,943 (8%)
Coronary artery disease	3,676 (14%)
Hypertension	12,581 (49%)
Current smoker	6,696 (26%)
Cholesterol (mg/dl)	
Total	223 (48)
LDL	140 (42)
HDL	53 (15)
Triglycerides (mg/dl)	66 (44)

Supplementary Table 15. Model fit for lipid polygenic risk score modeling in HUNT.

LDPred model

rho	N SNPs	R2
Infinitesimal	2,013,592	0.212114156
1.00	2,013,592	0.210936363
0.30	2,013,592	0.219802889
0.10	2,013,592	0.241752015
0.03	2,013,592	0.276071541
0.01	2,013,592	0.298016009
0.003	2,013,592	0.196639074
0.001	2,013,592	0.194917519
0.0003	2,013,592	0.191353777
0.0001	2,013,592	0.217751353
Unweighted	2,013,592	0.206473811

LD pruning / p-value thresholding

r^2	p-value	N SNPs	R2
0.2	1.00	191,041	0.212253965
0.2	0.50	130,237	0.213372646
0.2	$5x10^{-2}$	20,455	0.234203378
0.2	$5x10^{-4}$	1,105	0.273260701
0.2	$5x10^{-6}$	435	0.270072666
0.2	$5x10^{-8}$	282	0.267177166
0.4	1.00	303,609	0.215066578
0.4	0.50	191,884	0.216963972
0.4	$5x10^{-2}$	25,779	0.2440181
0.4	$5x10^{-4}$	1,457	0.274625719
0.4	$5x10^{-6}$	615	0.270472212
0.4	$5x10^{-8}$	411	0.268238831
0.6	1.00	431,507	0.215830911
0.6	0.50	254,486	0.218009208
0.6	$5x10^{-2}$	31,494	0.245994819
0.6	$5x10^{-4}$	1,869	0.267250615
0.6	$5x10^{-6}$	819	0.263103684
0.6	$5x10^{-8}$	539	0.259650587
0.8	1.00	600,685	0.215212651
0.8	0.50	334,230	0.217733187
0.8	$5x10^{-2}$	394,38	0.244644548
0.8	$5x10^{-4}$	2,451	0.261998951
0.8	$5x10^{-6}$	1,090	0.258413457
0.8	$5x10^{-8}$	730	0.25478395
Restricted		59	0.245520743

We trained several polygenic risk scores, based on two models (LDPred[15], and LD pruning and p-value thresholds). The key LDPred tuning parameter is rho, which is the assumption of the proportion of causal SNPs in the set of training SNPs. We varied parameters specific to each model for LDL cholesterol within the HUNT dataset. We selected the model with the best fit (by R2) to bring forward to analysis; this was LDPred rho=0.01 and is highlighted in the table.

Supplementary Fig. 1. Principal components and admixture across individuals analyzed.

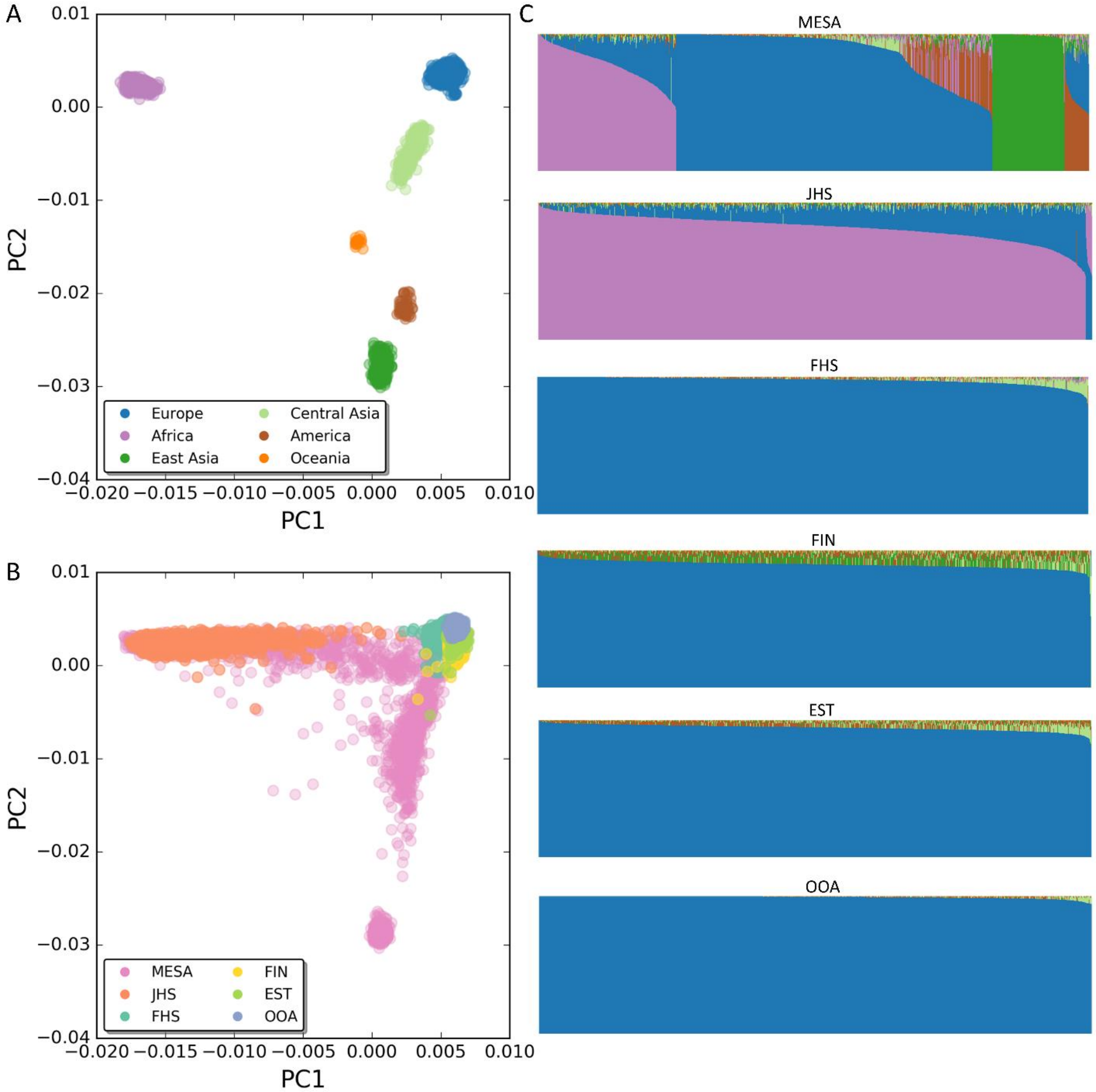

Approximately 16,000 ancestry-informative markers were used to estimate principle components of ancestry and admixture. **a.** Principal components of reference samples are displayed. **b.** Principal components of study samples are displayed. **c.** Ethnic admixture for all study samples by cohort are displayed.
EST = Estonia, FHS = Framingham Heart Study, FIN = Finland, JHS = Jackson Heart Study, MESA = Multi-Ethnic Study of Atherosclerosis, OOA = Old Order Amish

Supplementary Fig. 2. Distribution of whole genome sequencing coverage by cohort.

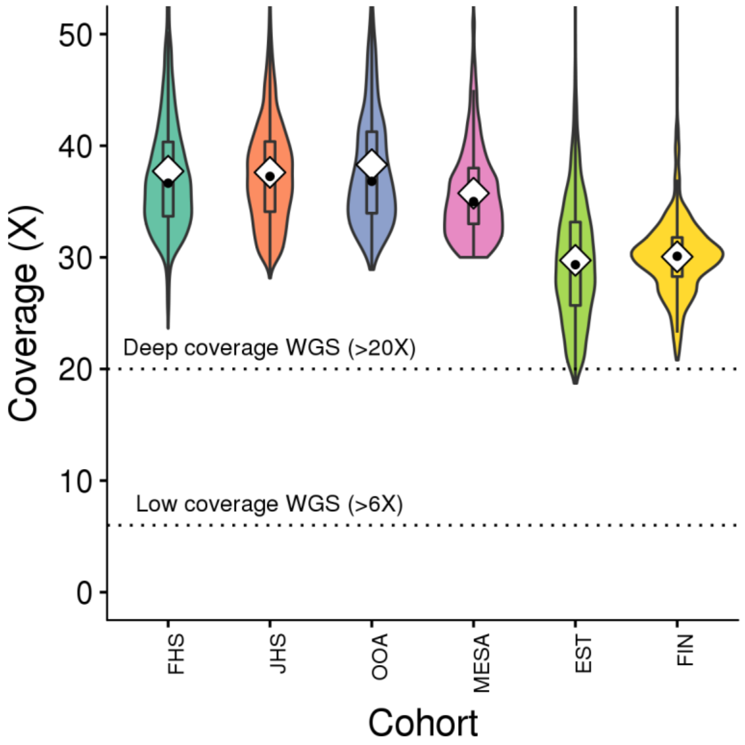

16,324 individuals underwent whole genome sequencing with target coverage >30X among FHS, JHS, OOA, and MESA participants, and >20X among EST and FIN participants.
EST = Estonia, FHS = Framingham Heart Study, FIN = Finland, JHS = Jackson Heart Study, MESA = Multi-Ethnic Study of Atherosclerosis, OOA = Old Order Amish

Supplementary Fig. 3. Quantile-quantile and Manhattan plots of single variant associations with blood lipids.

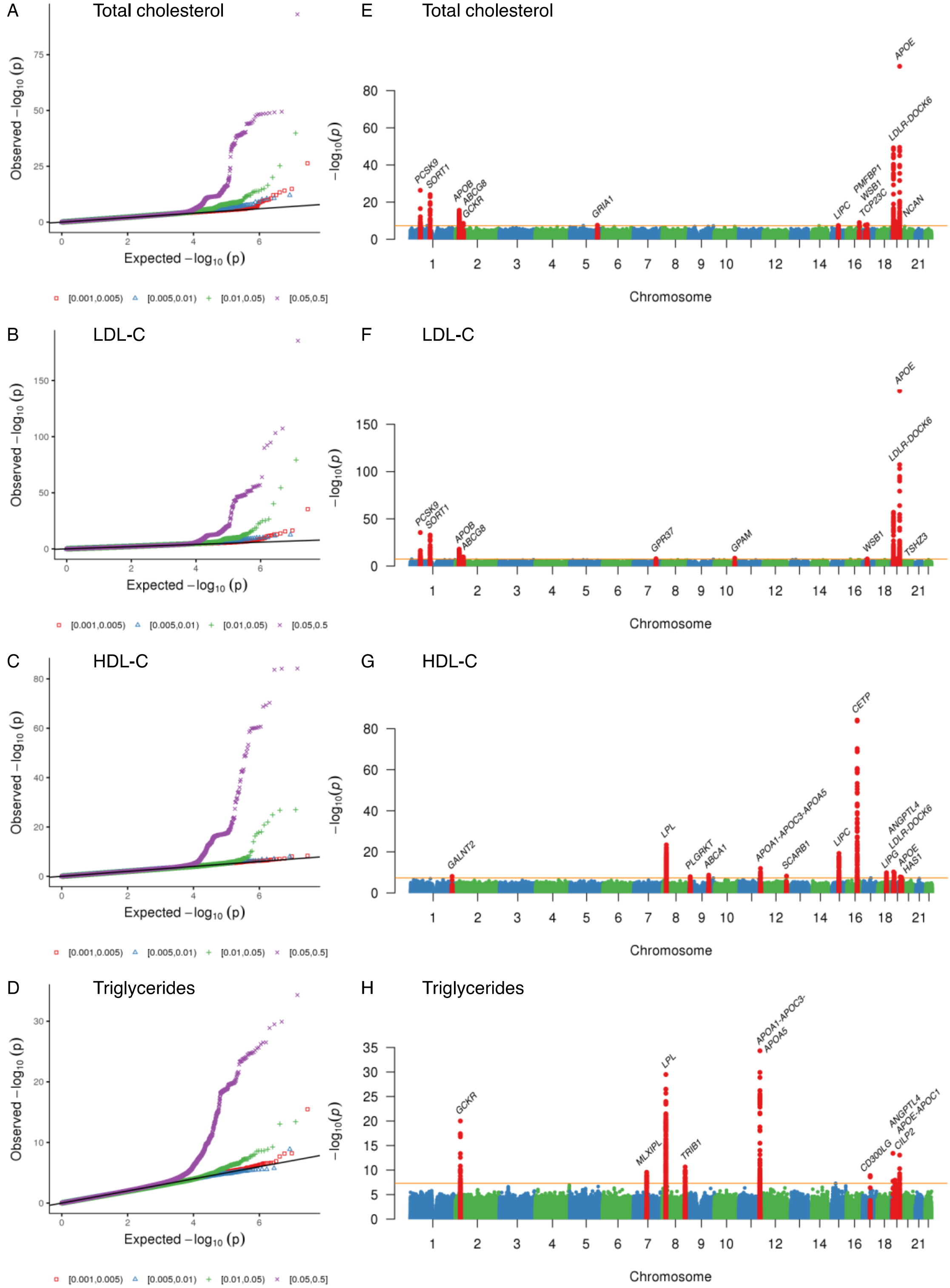

Individual variants were associated with total cholesterol, LDL-C, HDL-C, and triglycerides within three variant callsets and meta-analyzed. Variants that achieved >0.1% for a given callset were included in the meta-analysis.

Given that *APOB* p.R3527Q explains a large proportion of variance of total cholesterol and LDL-C among OOA, presence of this mutation was adjusted for in the TOPMed Phase 1 according analyses. Given that *APOC3* p.R19Ter explains a large proportion of variance of HDL-C and triglycerides among OOA, presence of this mutation was adjusted for in the TOPMed Phase 1 according analyses. **a.-d.** Quantile-quantile plots and **e.-h.** Manhattan plots are displayed.

HDL-C = high-density lipoprotein cholesterol, JHS = Jackson Heart Study, LDL-C = low-density lipoprotein cholesterol, OOA = Old Order Amish, TOPMed = Trans-Omics for Precision Medicine

Supplementary Fig 4. Association of 9p24.1 locus 1-bp deletion with HDL cholesterol.

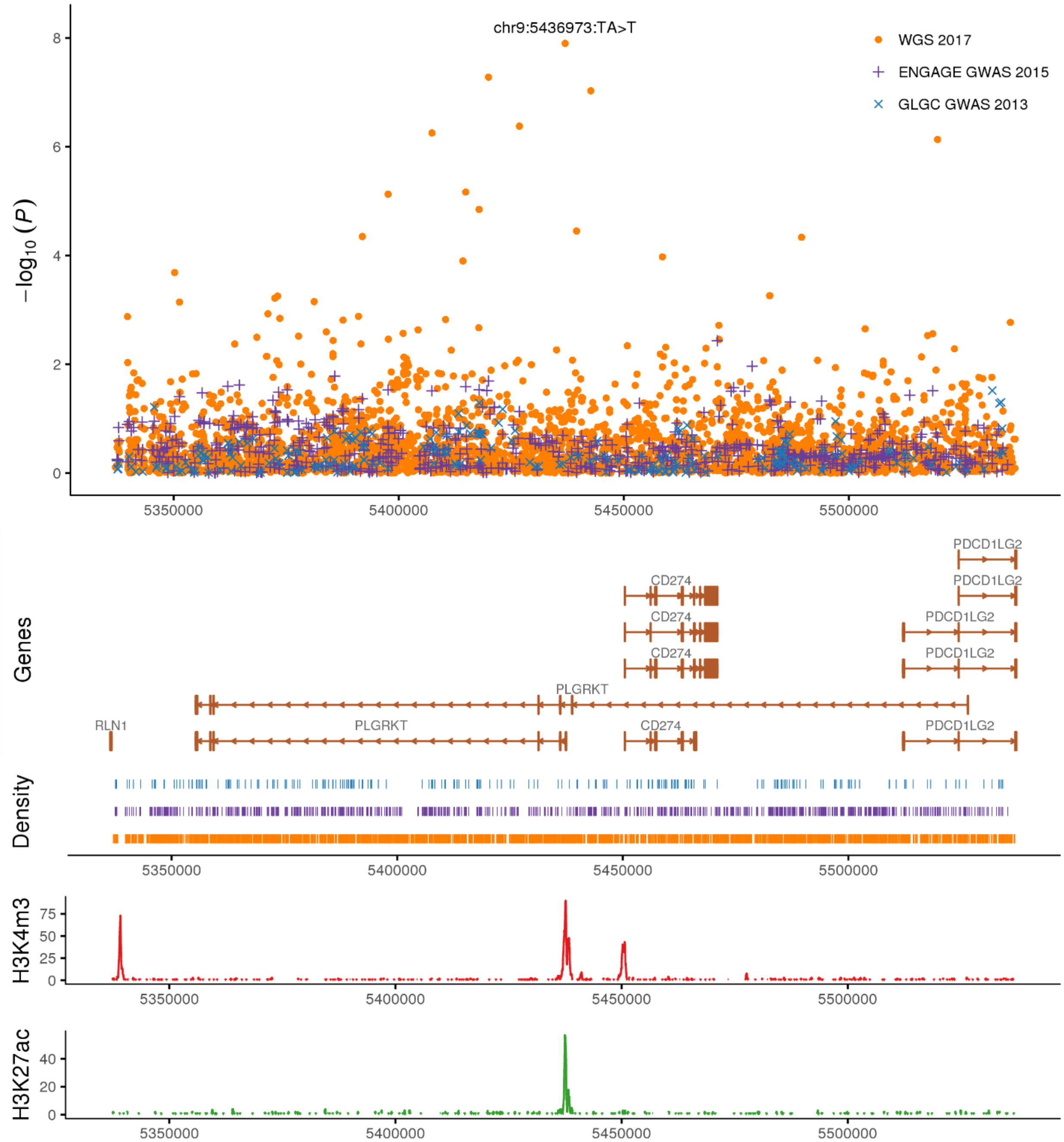

Regional association of meta-analysis $-\log_{10}$(P) results at the 9p24.1 locus for association with HDL-C are presented with prior genome-wide association analyses with array-derived genotypes. The index 1-bp deletion is not in linkage disequilibrium with variants previously catalogued by prior analyses. This variant lies within an intron of *PLGRKT* and overlies H3K4m3 and H3K27ac chromatin modifications, indicating promoter and enhancer regions, respectively.

Supplementary Fig 5. African American-specific haplotype at the *LDLR* locus associated with LDL cholesterol.

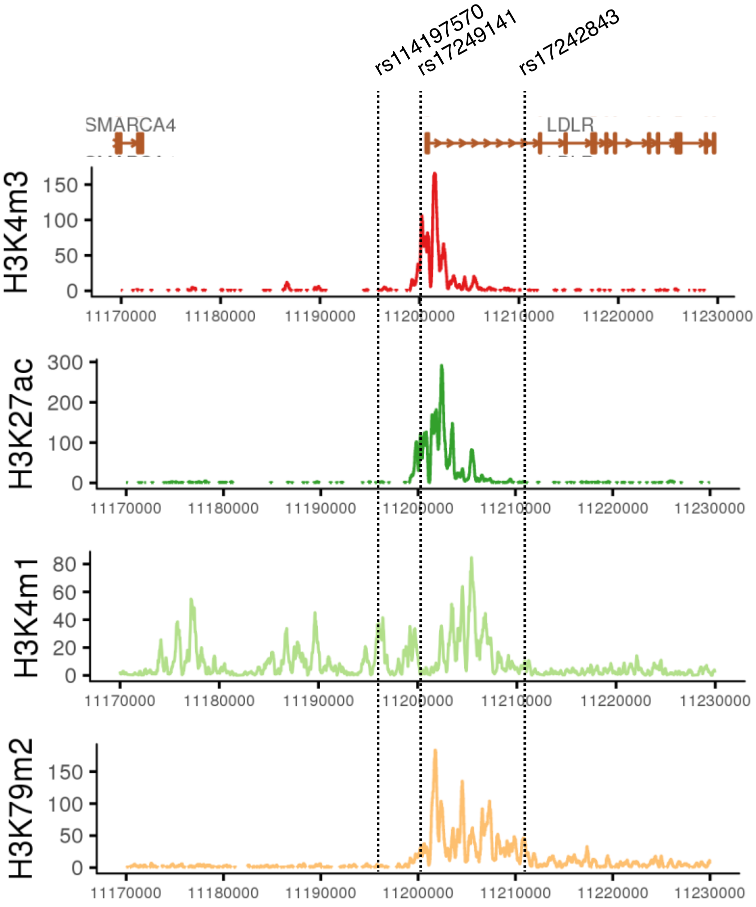

Independent variants common among African Americans in linkage disequilibrium ($r^2 > 0.8$) at the *LDLR* locus associated with LD-C. Standardized histone modification scores for HepG2 cells are displayed, including H3K4m3 (active promoter), H3K27ac (strong enhancer), H3K4m1 (weak enhancer), and H3K79m2 (transcription transition).

Supplementary Fig. 6. Haplotype structure of 5' *LDLR* variants associated with LDL cholesterol.

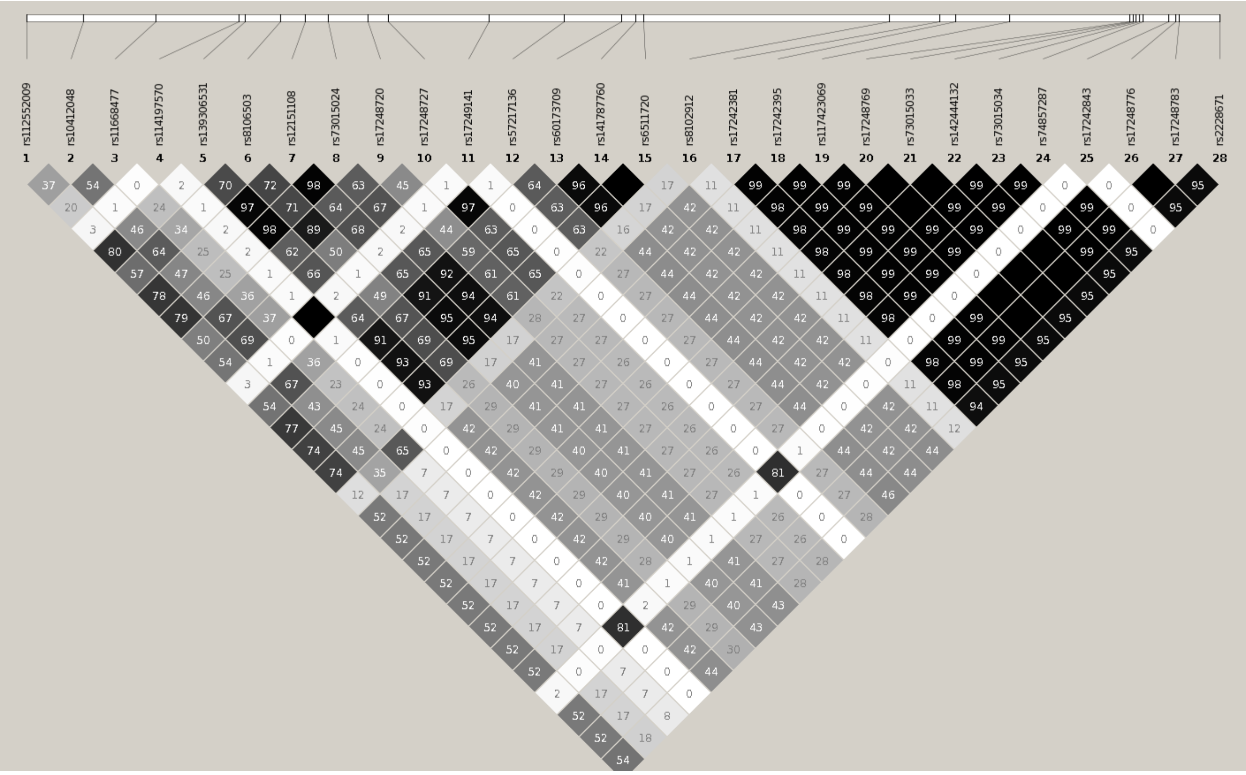

Haplotype of variants at chr19:11,193,080-11,211,000 (hg19), MAF > 0.1% within the TOPMed Phase I samples associated with LDL-C($P < 1 \times 10^{-10}$).

MAF = minor allele frequency; LDL-C = low-density lipoprotein cholesterol; TOPMed = Trans-Omics for Precision Medicine

Supplementary Fig. 7. Regional association plot at the *LDLR* locus highlighting an African American-specific haplotype associated with LDL cholesterol.

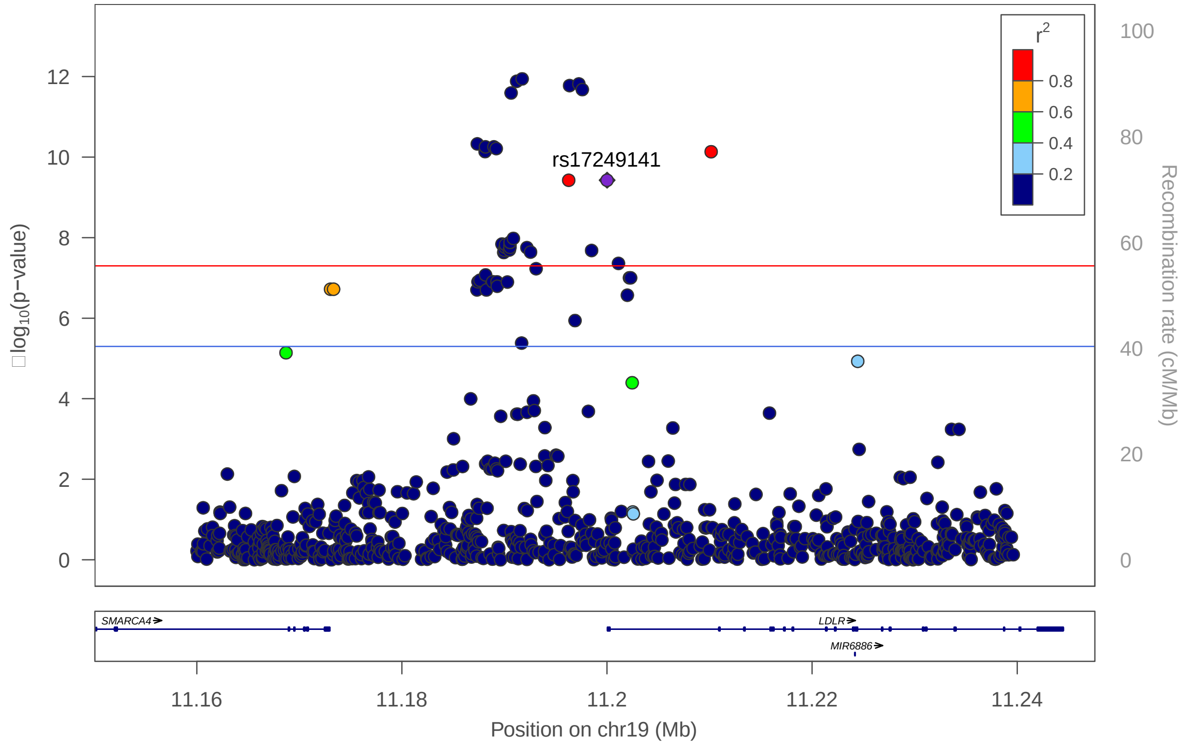

Variants at the *LDLR* locus associated with LDL-C among the TOPMed Phase I samples. An African American-specific associated haplotype is highlighted (purple and red).
LDL-C = low-density lipoprotein cholesterol; TOPMed = Trans-Omics for Precision Medicine

Supplementary Fig. 8. Combined minor allele frequency of disruptive mutations per gene.

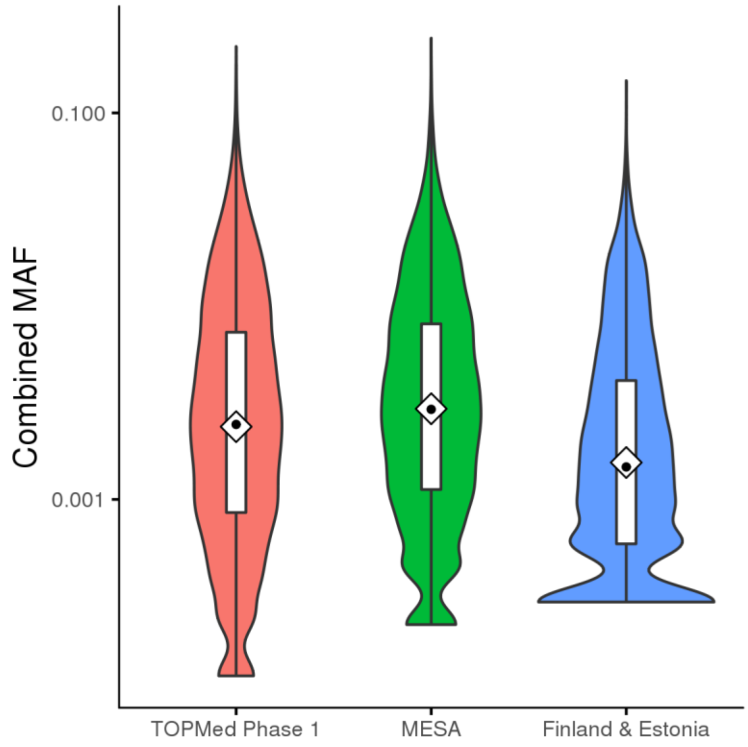

Rare (MAF < 1%) variants annotated as loss-of-function or disruptive missense mutations by MetaSVM[16] were identified. The distribution of combined MAF of these variants per gene are displayed. MAF = minor allele frequency, MESA = Multi-Ethnic Study of Atherosclerosis, TOPMed = Trans-Omics for Precision Medicine

Supplementary Fig. 9. Quantile-quantile plots of rare variant association tests.

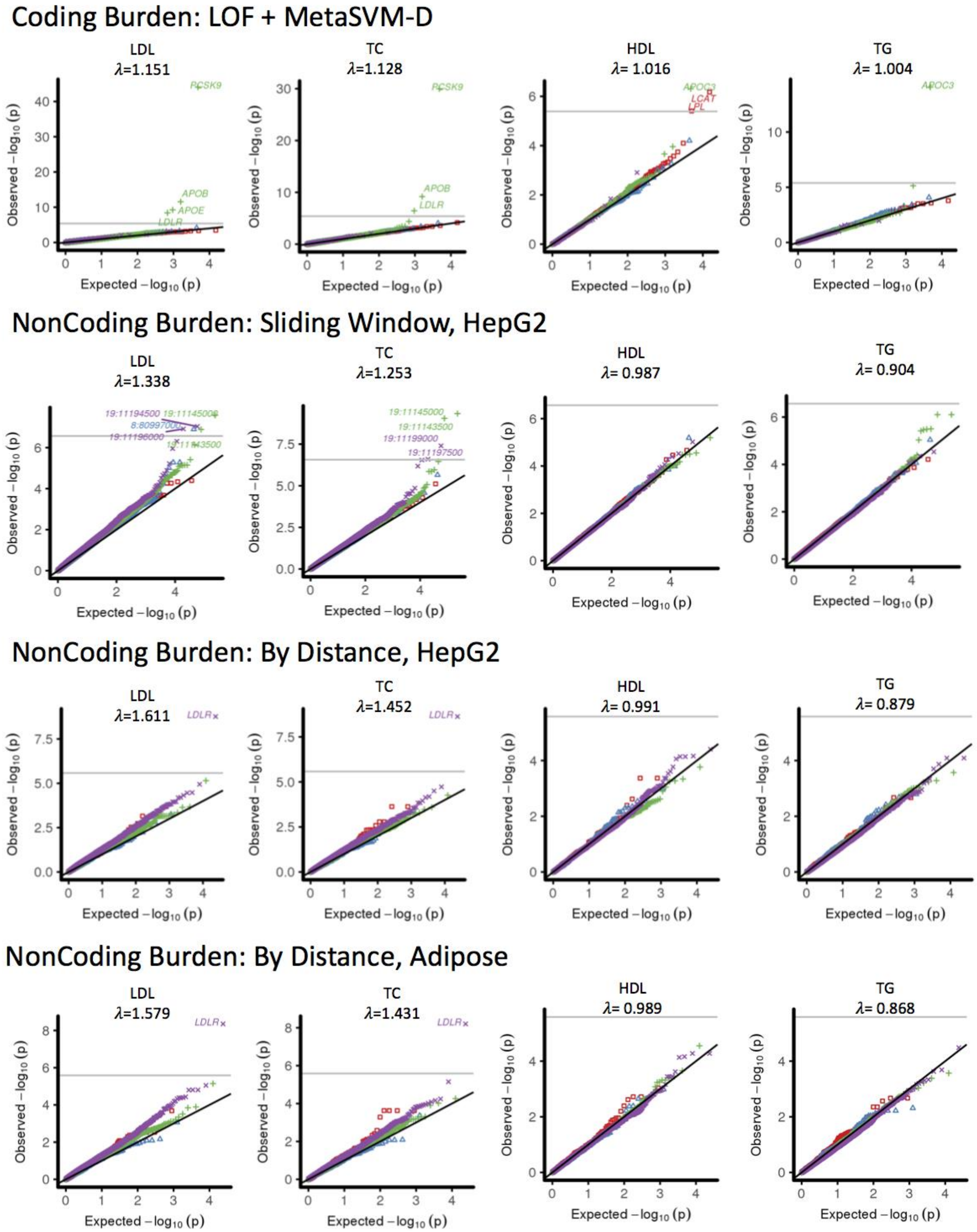

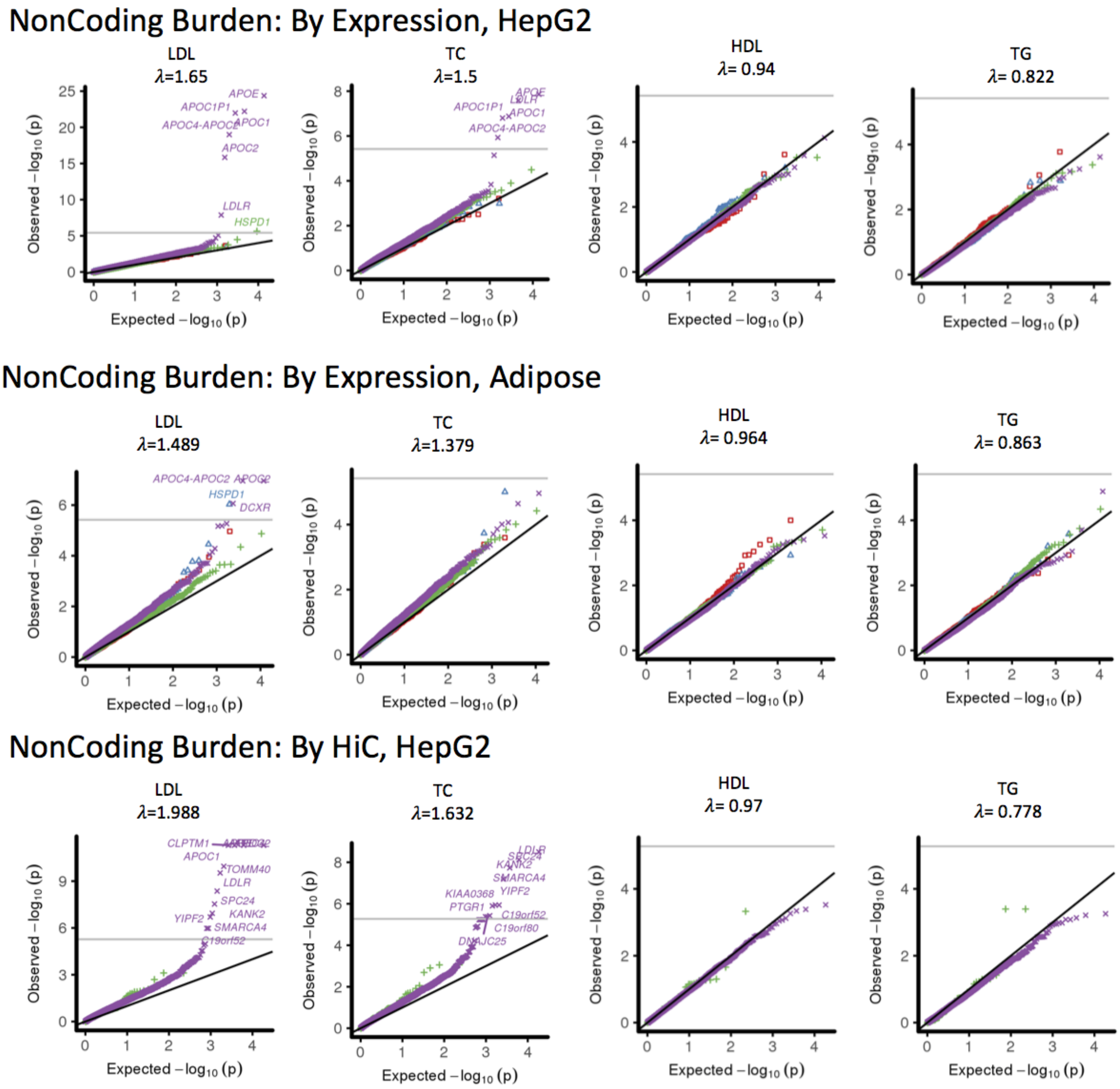

Genome-wide rare variant coding and non-coding association analyses are presented for total cholesterol, HDL-C, LDL-C, and triglycerides. Dots are color coded by combined MAF bin (purple: MAF > 5%, green: MAF 1-5%, blue MAF 0.5-1%, red MAF 0.1-0.5%). Horizontal line corresponds to –log10 of Bonferroni-corrected alpha threshold: 0.05/20,000 gene-associated groups = 2.5×10^{-6}.

HDL-C = high-density lipoprotein cholesterol; LDL-C = low-density lipoprotein cholesterol; LOF = loss-of-function; MAF = minor allele frequency; TC = total cholesterol; TG = triglycerides

Supplementary Fig. 10. Coding and non-coding rare variant association analyses for *LDLR*.

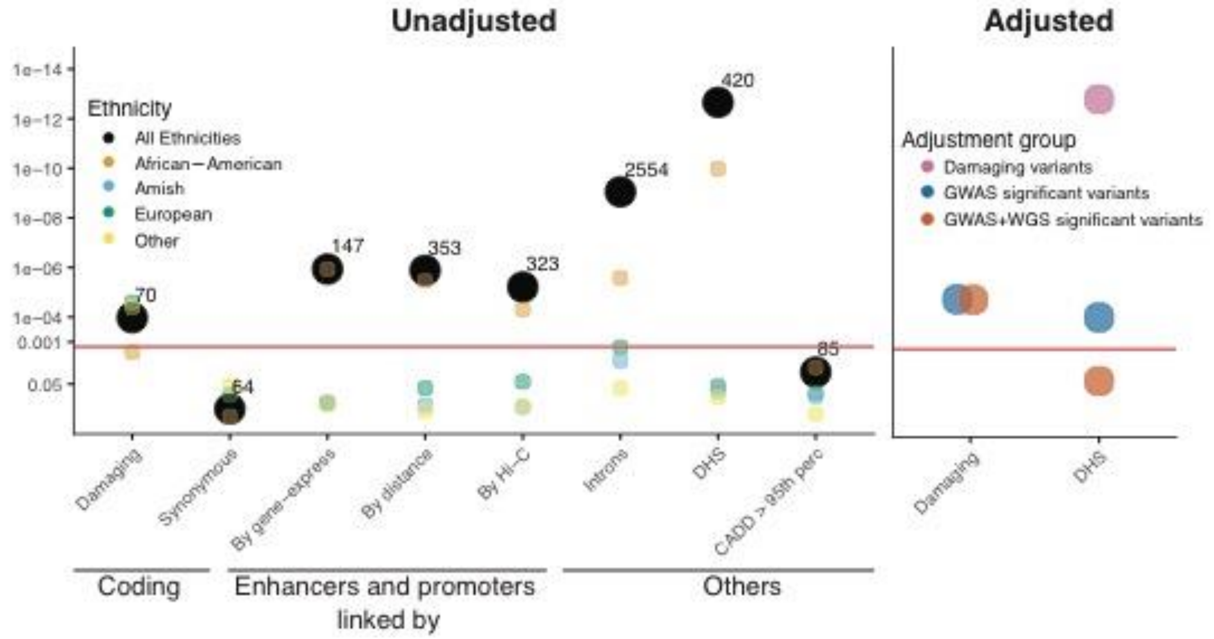

Results of coding and non-coding rare (MAF < 1%) variant testing are presented. Numbers represent the number of variants included in grouping strategies. The line represents the Bonferroni level of significance ($P = 0.05 / 13$ tests $= 0.004$). Damaging coding variants remain associated despite conditioning on single variants reaching genome-wide significance either previously or in our study ($P < 5x10^{-8}$). After observing the strongest non-coding association with DNase hypersensitivity sites, we did additional conditional analyses. Non-coding association was independent of coding associations but was no longer present after adjusting for individually-associated ($P < 5x10^{-8}$) variants.

Supplementary References

1. Kannel, W.B., Dawber, T.R., Kagan, A., Revotskie, N. & Stokes, J., 3rd. Factors of risk in the development of coronary heart disease--six year follow-up experience. The Framingham Study. *Ann Intern Med* **55**, 33-50 (1961).
2. Taylor, H.A., Jr. *et al.* Toward resolution of cardiovascular health disparities in African Americans: design and methods of the Jackson Heart Study. *Ethn Dis* **15**, S6-4-17 (2005).
3. Post, W. *et al.* Determinants of coronary artery and aortic calcification in the Old Order Amish. *Circulation* **115**, 717-24 (2007).
4. Mitchell, B.D. *et al.* The genetic response to short-term interventions affecting cardiovascular function: rationale and design of the Heredity and Phenotype Intervention (HAPI) Heart Study. *Am Heart J* **155**, 823-8 (2008).
5. Bild, D.E. *et al.* Multi-Ethnic Study of Atherosclerosis: objectives and design. *Am J Epidemiol* **156**, 871-81 (2002).
6. Vartiainen, E. *et al.* Cardiovascular risk factor changes in Finland, 1972-1997. *Int J Epidemiol* **29**, 49-56 (2000).
7. Nelis, M. *et al.* Genetic structure of Europeans: a view from the North-East. *PLoS One* **4**, e5472 (2009).
8. Kathiresan, S. *et al.* Common variants at 30 loci contribute to polygenic dyslipidemia. *Nat Genet* **41**, 56-65 (2009).
9. Surakka, I. *et al.* The impact of low-frequency and rare variants on lipid levels. *Nat Genet* **47**, 589-97 (2015).
10. Teslovich, T.M. *et al.* Biological, clinical and population relevance of 95 loci for blood lipids. *Nature* **466**, 707-13 (2010).
11. Willer, C.J. *et al.* Discovery and refinement of loci associated with lipid levels. *Nat Genet* (2013).
12. Pulit, S.L., de With, S.A. & de Bakker, P.I. Resetting the bar: Statistical significance in whole-genome sequencing-based association studies of global populations. *Genet Epidemiol* **41**, 145-151 (2017).
13. Landrum, M.J. *et al.* ClinVar: public archive of interpretations of clinically relevant variants. *Nucleic Acids Res* **44**, D862-8 (2016).
14. Karczewski, K.J. LOFTEE (Loss-Of-Function Transcript Effect Estimator). (2015).
15. Vilhjalmsson, B.J. *et al.* Modeling Linkage Disequilibrium Increases Accuracy of Polygenic Risk Scores. *Am J Hum Genet* **97**, 576-92 (2015).
16. Dong, C. *et al.* Comparison and integration of deleteriousness prediction methods for nonsynonymous SNVs in whole exome sequencing studies. *Hum Mol Genet* **24**, 2125-37 (2015).
