# Supplementary material for: Deep-coverage whole genome sequences and blood lipids among 16,324 individuals
Source: Nat Commun. 2018 Aug 23;9:3391. doi: 10.1038/s41467-018-05747-8 (PMC6107638; doi:10.1038/s41467-018-05747-8)
Supplement: Supplementary file 2 — Peer Review File [file 41467_2018_5747_MOESM2_ESM.pdf]

Editorial Note: this manuscript has been previously reviewed at another journal that is not operating a transparent peer review scheme. This document only contains reviewer comments and rebuttal letters for versions considered at Nature Communications.

Reviewer #1 (Remarks to the Author):

Thanks for asking me to review this revised manuscript following the authors revisions and the switch from NG to NC. The authors have done a great job of responding to the comments that i (and the other reviewers) raised on the previous version. I have no further comments.

Reviewer #2 (Remarks to the Author):

The authors have made a good faith effort to respond to reviewer comments, with new analyses and some new text.

All responses are satisfactory. The paper's reach now does not exceed its grasp.

I have no further comments.

Response to Reviewers

Reviewer #1:

Thanks for asking me to review this revised manuscript following the authors revisions and the switch from NG to NC. The authors have done a great job of responding to the comments that I (and the other reviewers) raised on the previous version. I have no further comments.

We appreciate your thoughtful review.

Reviewer #2:

The authors have made a good faith effort to respond to reviewer comments, with new analyses and some new text.

All responses are satisfactory. The paper's reach now does not exceed its grasp.

I have no further comments.

We appreciate your thoughtful review.
